# Emergency department syndromic surveillance systems: a systematic review

**DOI:** 10.1186/s12889-020-09949-y

**Published:** 2020-12-09

**Authors:** Helen E. Hughes, Obaghe Edeghere, Sarah J. O’Brien, Roberto Vivancos, Alex J. Elliot

**Affiliations:** 1 Real-time Syndromic Surveillance Team, Field Service, National Infection Service, Public Health England, Birmingham, UK; 2 Farr Institute@HeRC, University of Liverpool, Liverpool, UK; 3 Field Epidemiology West Midlands, Field Service, National Infection Service, Public Health England, Birmingham, UK; 4 School of Natural and Environmental Sciences, Newcastle University, Newcastle, UK; 5 Field Epidemiology North West, Field Service, National Infection Service, Public Health England, Liverpool, UK

**Keywords:** Syndromic surveillance, Emergency department, Public health, Acute illness, Emergency room, Accident and emergency, Real-time surveillance, Outbreak, Terrorism, Natural disaster

## Abstract

**Background:**

Syndromic surveillance provides public health intelligence to aid in early warning and monitoring of public health impacts (e.g. seasonal influenza), or reassurance when an impact has not occurred. Using information collected during routine patient care, syndromic surveillance can be based on signs/symptoms/preliminary diagnoses. This approach makes syndromic surveillance much timelier than surveillance requiring laboratory confirmed diagnoses.

The provision of healthcare services and patient access to them varies globally. However, emergency departments (EDs) exist worldwide, providing unscheduled urgent care to people in acute need. This provision of care makes ED syndromic surveillance (EDSyS) a potentially valuable tool for public health surveillance internationally.

The objective of this study was to identify and describe the key characteristics of EDSyS systems that have been established and used globally.

**Methods:**

We systematically reviewed studies published in peer review journals and presented at International Society of Infectious Disease Surveillance conferences (up to and including 2017) to identify EDSyS systems which have been created and used for public health purposes. Search criteria developed to identify “emergency department” and “syndromic surveillance” were applied to *NICE healthcare, Global Health* and *Scopus* databases.

**Results:**

In total, 559 studies were identified as eligible for inclusion in the review, comprising 136 journal articles and 423 conference abstracts/papers. From these studies we identified 115 EDSyS systems in 15 different countries/territories across North America, Europe, Asia and Australasia. Systems ranged from local surveillance based on a single ED, to comprehensive national systems. National EDSyS systems were identified in 8 countries/territories: 2 reported inclusion of ≥85% of ED visits nationally (France and Taiwan).

**Conclusions:**

EDSyS provides a valuable tool for the identification and monitoring of trends in severe illness. Technological advances, particularly in the emergency care patient record, have enabled the evolution of EDSyS over time. EDSyS reporting has become closer to ‘real-time’, with automated, secure electronic extraction and analysis possible on a daily, or more frequent basis.

The dissemination of methods employed and evidence of successful application to public health practice should be encouraged to support learning from best practice, enabling future improvement, harmonisation and collaboration between systems in future.

**Prospero number:**

CRD42017069150.

**Supplementary Information:**

The online version contains supplementary material available at 10.1186/s12889-020-09949-y.

## Background

Syndromic surveillance is a relatively recent addition to the public health surveillance toolbox, with the earliest reported systems established during the mid-1990s [1]. Syndromic surveillance uses symptom and/or preliminary diagnosis information and rapid data collection methods to provide information for public heath action. Syndromic surveillance is more timely than other more traditional options for public health surveillance, such as statutory notifications of disease or laboratory reporting [2]. The non-specific nature of syndromic surveillance and its rapid data collection also makes it sensitive and flexible enough to respond to different situations/scenarios including infectious outbreaks and non-infectious disease events. The data used for syndromic surveillance are primarily gathered from patient contacts with a health care service, although increasingly non-health care syndromic surveillance data are being explored e.g. social media [3] or internet search data [4, 5].

The sources of patient health information used for syndromic surveillance are as varied as the different types of health care provision that exist. Examples of syndromic surveillance data range from; calls from those who are ill in the community to telehealth advice phone lines [6, 7], to patients attending in person in primary care (family doctors) [8, 9], or in emergency care situations including emergency departments (ED).

Patients seen in the ED are generally expected to be presenting with severe illness requiring immediate, often lifesaving, medical attention and treatment. This severe level of acute illness is of particular interest to public health surveillance to enable the identification and monitoring of public health issues requiring an acute response. Conversely, this surveillance may also provide reassurance, confirming that there is no public health impact from an incident already identified.

Healthcare systems vary, however, EDs are commonly found worldwide, providing unscheduled emergency care to patients as required. The global presence of EDs has facilitated the increasing use of ED clinical data for syndromic surveillance purposes. To date there has not been a review of the ED systems developed worldwide, with only one systematic review on the use of ED syndromic surveillance (EDSyS) for influenza [10].

Here, we systematically review the available literature to identify and describe the range of EDSyS systems reported to have been developed for public health use globally. We describe the different models developed to collect and analyse ED data, and the public health uses of EDSyS. Additionally, we discuss the changes and development of these systems over time and the potential for future development.

## Methods

This systematic review was carried out following the Preferred Reporting Items for Systematic Reviews and Meta-Analyses (PRISMA) [11] and was registered on Prospero [12], reference number: CRD42017069150.

### Identification of studies

Searches were carried out using the NICE healthcare database (HDAS, accessing *PubMed*, *MedLine*, *EmBase*, *Health Business Elite*, *Health Management Information Consortium*, *PsycINFO*, *British Nursing Index*, and *Cumulative Index to Nursing* and *Allied Health Literature*), in addition to the *Global Health* (accessed through *EBSCO*) and *Scopus* online databases.

Search terms were developed to identify published papers demonstrating an operational EDSyS system collecting, analysing and reporting in near real-time for public health purposes. These papers required inclusion of terms related to both syndromic surveillance AND to ED, in the title and/or abstract. The electronic HDAS search string was:

(“emergency department” OR “emergency room” OR “emergency care” OR “emergency medical” OR “chief complaint” OR “presenting complaint” OR “triage”) AND (“syndromic surveillance” OR “real-time surveillance” OR “real time surveillance” OR “syndrome surveillance”).ti,ab.

Where review-type studies were identified, the references from each were searched to identify any primary research studies describing an eligible system not identified elsewhere during the search.

The restriction to English language peer reviewed publications was recognised as a possible bias against ED systems established in non-English speaking countries/territories, or smaller systems which may not be written up for formal publication. In order to counteract this potential selection bias, all available abstracts/papers for the International Society for Disease Surveillance (ISDS) annual conferences up to 2016 were also included in the search (including predecessor conferences, beginning 2002: no conference was held in 2017). ISDS conference abstracts which included the eligible search terms were identified through searching of conference abstract archives available in online journals [13–26]. Abstracts for the 2009 conference were obtained through personal communication with ISDS as an online archive was not available.

### Included studies

We included all studies which included reference to an operational EDSyS system, defined as an EDSyS which collected, analysed and reported on ED data in real time, for public health purposes. The search was limited to studies published up to and including 31 December 2017, with no limitation on the search start date.

### Excluded studies

We excluded studies reporting on the use of retrospectively accessed ED data from a source other than an operational EDSyS system (e.g. directly from an ED information system or other database). These retrospective studies were generally investigated the potential use and/or benefits of ED data for syndromic surveillance purposes. Non-English language journal articles were excluded, as were book chapters, non ISDS conference abstracts/papers, dissertations and reports.

### Screening

The selection of studies for inclusion was carried out independently by two reviewers (HEH and OE) using Covidence [27]. All titles and abstracts were initially screened to identify only those which reported on, or appeared to report on, an operational EDSyS system. Full text screening was carried out by both reviewers selecting studies that met the inclusion criteria. Any conflicts were resolved by HEH.

### Data extraction

Following full text screening, studies meeting selection criteria were then subjected to qualitative data extraction. The data extracted included: EDSyS location; motivation for system creation; system start date; coverage; and the dates and coverage of any research project reported. Where available, information was also extracted describing: the technical details of the system (timings, frequency and methods of data collection and transfer of data from the ED to the syndromic surveillance database). Qualitative data details included: the syndromic indicators used (data source, format and syndromes of interest); the analytical techniques used; and public health actions carried out in response to the surveillance findings.

Data extraction from all studies was carried out by the primary reviewer (HEH). The secondary reviewer (OE) undertook a quality control check by extracting information from a random 10% sample of studies.

## Results

**Fig. 1:**
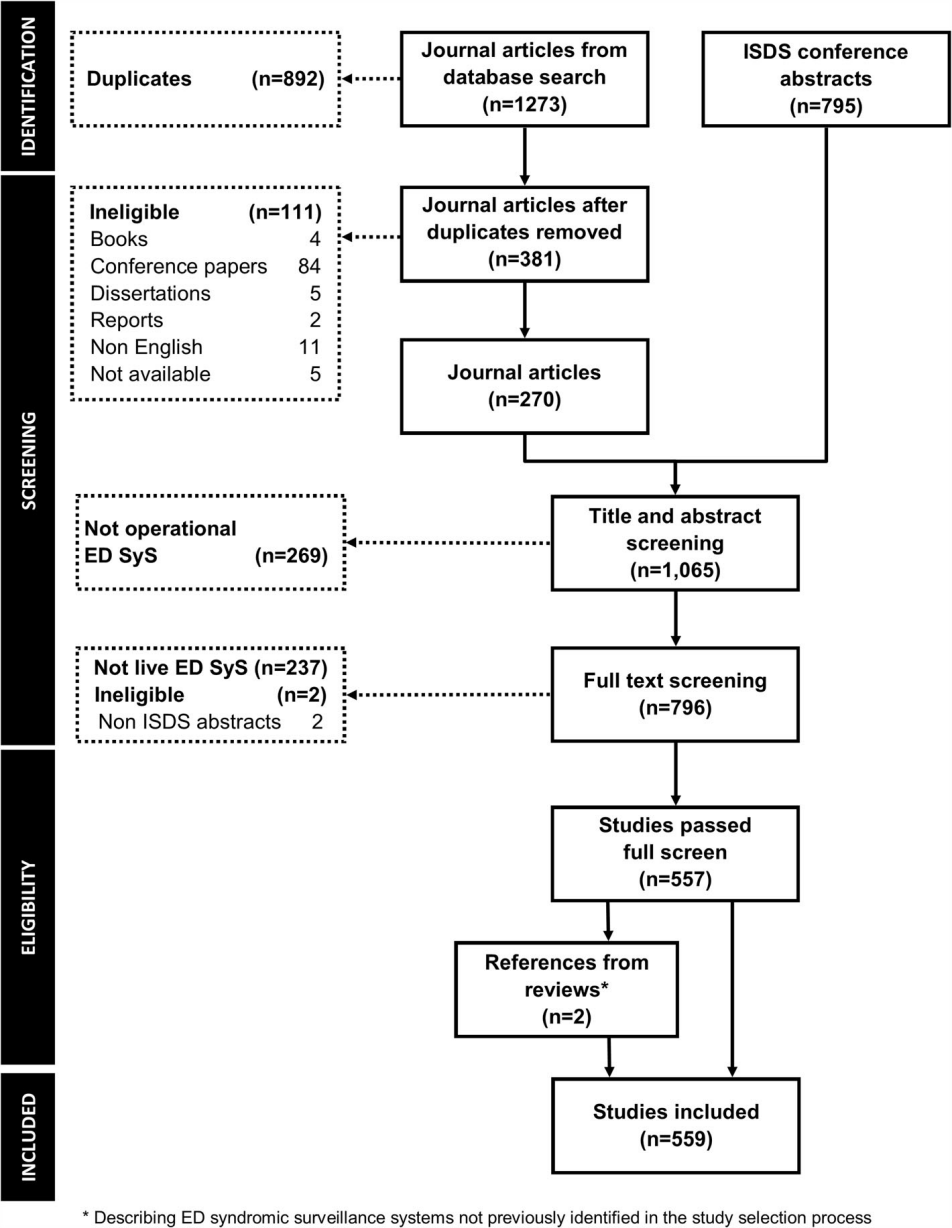
PRISMA flow diagram of the screening process and numbers of articles identified

In total 1273 journal articles were identified, with publication dates from 2002 to 2017. Duplicate (*n* = 892) and articles not eligible for inclusion (*n* = 111) were removed. Additionally, 795 ISDS conference abstracts were identified for inclusion. Of these the full conference papers were available for conferences held in 2003 and 2004 (Fig. 1).

Title and abstract screening of the resulting 1065 studies (270 journal articles and 795 ISDS conference abstracts/papers) excluded 237 studies that did not clearly describe an operational EDSyS system (or the use of data from one) and two studies that were identified as non-ISDS conference abstracts. The resulting 796 studies were included for full text screening (Fig. 1).

**Fig. 2:**
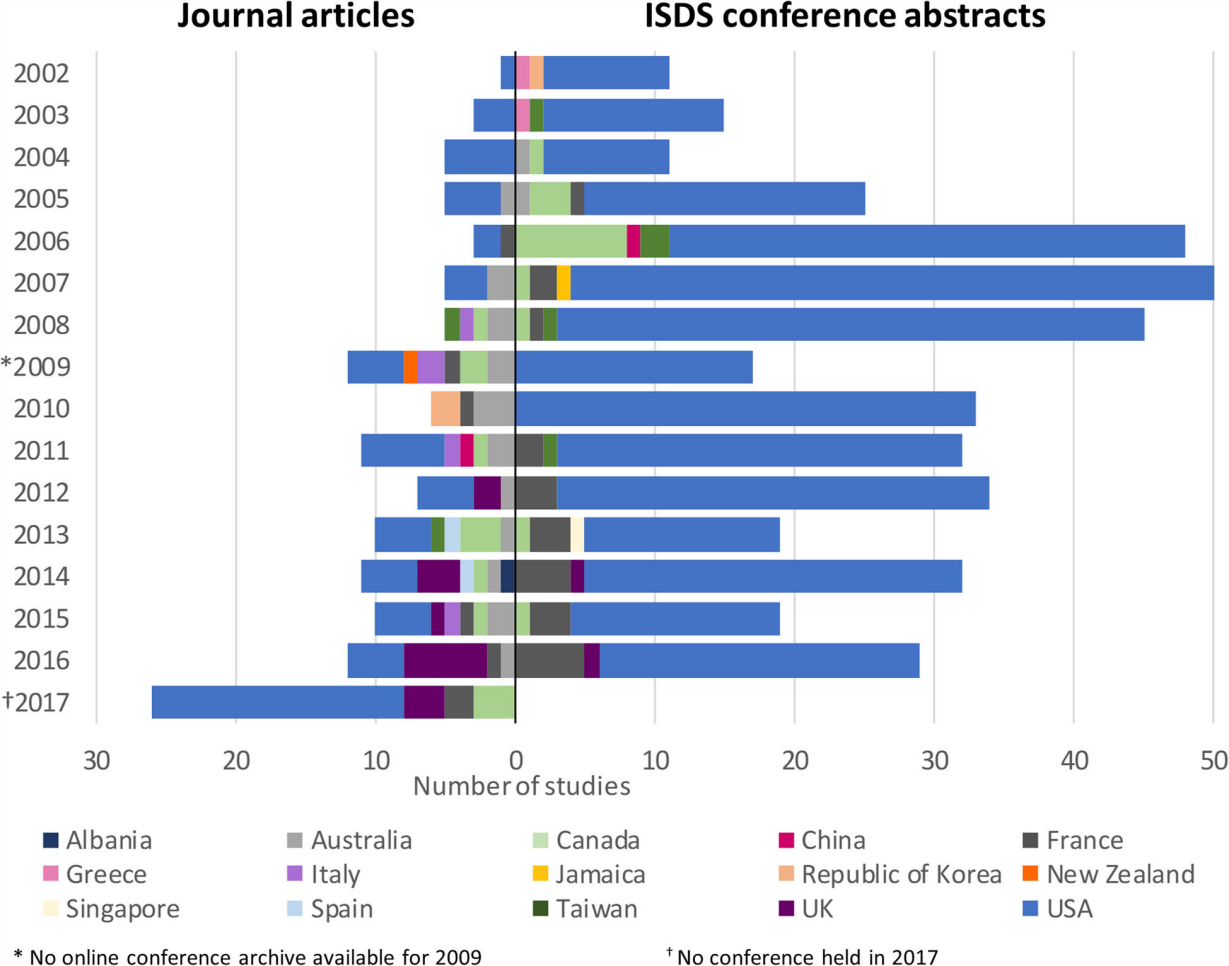
Number of journal articles and International Society for Disease Surveillance (ISDS) conference abstracts identified, by year of publication/conference and country/territory

The full text screen identified one systematic review [10], one case study of three separate EDSyS systems [28] and one review of automated outbreak detection in syndromic surveillance systems (not limited to EDSyS) [29]. These three manuscripts included description of multiple EDSyS systems, two of which had not been identified by the original search. These two additional EDSyS systems had primary references, which were added to the full data extraction ( Fig. 2).

In total 559 studies were identified as eligible for inclusion in the review comprising 136 journal articles and 423 ISDS conference abstracts/papers. A full list of all references is included in this review are available in (Additional file 1), which includes a detailed summary of all EDSyS systems identified, by country/territory, with sub national breakdown where appropriate.

The eligibility criteria allowed for individual EDSyS systems to be described in multiple references. The quality control check with the second reviewer extracting did not reveal any errors.

### Summary of global EDSyS

Each EDSyS included in the review had a single underlying aim to provide information for public health action. This aim encompassed the use of EDSyS in the monitoring of seasonal and sporadic, infectious and non-infectious disease activity, as well as the detection and the monitoring of the impact of unusual/unanticipated events (including natural disasters and bioterrorism).

The descriptions of EDSyS systems identified in the review were grouped by country/territory in order to summarise the reporting of the large number of systems. The following description of findings is based on this grouping, with individual examples highlighted as appropriate. A full list of EDSyS systems identified in the review is provided in Additional file 1.

The 559 studies included from the full screen comprised 115 EDSyS systems, in 15 countries and territories, across North America, Europe, Asia and Australasia (Table 1). The first EDSyS systems identified were all in the United States of America (USA), with four reported to have started data collection in 1999 [30–33] and a fifth reported in a study using data from 1999 [34].

**Table 1:** Summary of Emergency Department Syndromic Surveillance (EDSyS) systems by country/territory, detailing the number of systems, start date, coverage, initial design aim and the number (and type) of studies identified in this review

Country/territory	Number of systems	Earliest start year^a^	Coverage of individual system(s)		Initial design of system(s)		Number of studies identified^b^	
			National	Subnational	Aim	Event type	Journal papers	ISDS abstracts
Albania	1	2013^a^	**✓**	–	*Standard surveillance*	*No specific event*	1	0
Australia	3	2003	–	**✓**	Preparation	Mass gathering (sport)	18	2
					*Standard surveillance*	*No specific event*		
Canada	19	2002	–	**✓**	Preparation	Mass gathering (religious)	14	18
					*Standard surveillance*	*No specific event*		
China	2	2004^a^	–	**✓**	**Response**	**Outbreak (SARS)**	0	2
France	1	2004	**✓**	–	**Response**	**Natural disaster (heatwave)**	7	25
Greece	1	2002	–	**✓**	Preparation	Mass gathering (sport)	0	2
Italy	2	2000	–	**✓**	*Standard surveillance*	*No specific event*	5	0
Jamaica	1	2007	**✓**	–	Preparation	Mass gathering (sport)	0	1
Republic of Korea	1	2002	**✓**	–	Preparation	Mass gathering (sport)	2	1
					*Standard surveillance*	*No specific event*		
New Zealand	1	2008	–	**✓**	*Standard surveillance*	*No specific event*	2	0
Singapore	1	2013^a^	**✓**	–	–	–	0	1
Spain	1	2010^a^	–	**✓**	*Standard surveillance*	*No specific event*	2	0
Taiwan	2	2003	**✓**	**✓**	**Response**	**Outbreak (SARS)**	3	5
UK^c^	1	2010	**✓**	–	Preparation	Mass gathering (sport)	15	3
USA	78	1999	**✓**	**✓**	Preparation	Mass gathering (sport)	67	365
						Mass gathering (political)		
						Natural disaster (hurricane)		
					**Response**	**Terrorism**		
						**Outbreak (SARS)**		
					*Standard surveillance*	*Health surveillance requirements*		
						*No specific event*		

^a^ Start date not specified in all systems, so estimated from data used/ text ^b^ excluding reviews

^c^ UK: England & Northern Ireland **✓** EDSyS system in this category identified - no EDSyS system in this category/no information identified

EDSyS systems in four countries were identified solely from journal articles (Albania, Italy, New Zealand, Spain), whereas systems from three countries (Greece, Jamaica and Singapore) were identified only in ISDS conference abstracts/papers (Table 1**,** Fig. 2). Although the number of conference abstracts greatly outnumbered the journal articles, the number of journal articles published each year increased over time, from one in 2002, to 26 in 2017 (the only year during which there was no ISDS conference).

### Geographical coverage

ED services are a globally recognisable type of healthcare provision but access to these services and the administrative/organisational structures vary greatly. There is also variation in the organisation and delivery of public health services (delivered at national and sub-national levels), both between and within countries/territories. Each of these factors are likely to have impacted on the geographical and population coverage of EDSyS systems, which ranged from very local (including a single ED), to national systems, with many levels in between.

Six countries described having EDSyS systems developed with national coverage (Albania, France, Jamaica, Republic of Korea, Singapore, United Kingdom (UK): Table 1**,** Additional file 1). ‘National’ coverage varied in geographical (and consequently population) terms, with most being sentinel (Additional file 1). Where national ED systems had been developed, they were not solely used for national level investigation and reporting, with sub-national and localised geographical analyses also undertaken (e.g. overseas territories reported separately from France [35], and London reported from the UK [36]).

Seven countries had EDSyS systems working solely on a sub-national basis. Single, locally run systems were identified in Greece, New Zealand and Spain, whereas multiple stand-alone systems were identified in Australia, Canada, China and Italy (Table 1**,** Additional file 1).

EDSyS systems which had been separately developed at both national and sub-national levels were identified in Taiwan and USA (Table 1). The USA national system developments have been built upon (and subsequently extending) pre-existing local, sub-national EDSyS systems. Population-based systems were also identified in the USA, with dedicated military (including veteran) EDSyS operated at both state (North Carolina, in addition to a civilian EDSyS system) and national (potentially global) level (Additional file 1).

Descriptions of the EDSyS systems in both France and Taiwan reported ED participation to be ‘required’ (Taiwan) [37] and ‘mandatory’ (France) [38], with both reportedly receiving data from 85% or more of all ED visits (Additional file 1).

### The rationale for the development of EDSyS systems

This review identified three broad themes for EDSyS development and implementation. Firstly, EDSyS systems developed in preparation for an expected event (mass gathering or predictable natural disaster); secondly, those developed in response to an unanticipated event (natural disaster, outbreak or terrorism); or finally, EDSyS systems developed as a new standard surveillance format that was generally aimed to supplement and complement existing public health surveillance, adding resilience should any of the above events occur in future, including bioterrorism (Table 1).

In seven countries/territories, EDSyS systems were reportedly introduced in preparation for a mass gathering event (e.g. politics/religion/sport related), or even in advance of a predicted natural disaster (e.g. hurricane). A number of these systems were designed and run as short term, event-based systems, created shortly before and intended to be disbanded shortly after the event [30, 39–43]. Some of these short-lived event-based systems were subsequently redeveloped into ongoing operational EDSyS systems [30]. EDSyS systems created in preparation for a specific event, have also been intentionally designed from the outset to remain in place as standard surveillance capability, continuing as a legacy of the event [36, 44].

EDSyS systems developed in response to events of public health importance were implemented in response to infectious disease outbreaks (namely SARS [45–47]), terrorist events (September 2001 [48–51]) and natural disasters (heat wave [52]). The speed at which these systems were implemented was dependent on the level of immediate threat. Again, the design and structure of these systems may have been optimised for short lived surveillance (particularly when created quickly), but then further developed to become a routine surveillance system (e.g. New York City [53, 54]). Those responding to a non-immediate threat were created less rapidly as an ongoing, routine surveillance system (e.g. France [55]).

The creation of EDSyS systems solely to augment standard public health surveillance (including for the identification of bioterrorist threats) was identified as the primary purpose for the set-up of some systems, particularly small systems operating at a local level across the USA as well as others in New Zealand [56] and Republic of Korea [57].

### Data analysis

Real-time data collection and analysis on a more frequent than daily basis were described in systems from Australia [58], Canada [59] and the USA [60–62]. The analysis of EDSyS data was, however, most commonly reported to be conducted on a daily basis, even where data collection occurred more frequently [63–65].

The methods by which syndromic EDSyS data were analysed for exceedances or temporal spikes were often not clearly presented in the studies. The specific statistical methods applied to operational syndromic surveillance data in some studies were simply described as the use of ‘statistical algorithms’ or ‘aberration detection’. Statistical algorithms using or based on commonly used syndromic surveillance tools were reported in several EDSyS systems. This included reporting: the surveillance system used e.g. the Electronic Surveillance System for the Early Notification of Community-Based Epidemics (ESSENCE II) [66] or Real-time Outbreak and Disease Surveillance (RODS) [67]; the algorithm used e.g. Early Aberration Reporting System (EARS) [68]; or the tool used e.g. SatScan [69] for statistical analyses carried out. A bespoke statistical analysis method (Rising Activity, Multi-level Mixed effects, Indicator Emphasis, RAMMIE) was reported as a standard statistical method used for EDSyS in the UK [70].

Studies describing the development of statistical/analytical methods for use on syndromic data (rather than the application in day-to-day practice) were common. These studies focussed largely on the range of statistical methods and techniques that were available, proposals for potential statistical approaches and future developments [40, 71–87].

### Indicators monitored in EDSyS systems

Syndromic indicators were often-described for detecting ‘bioterrorist’ events [54, 88, 89]. Syndromic indicators were also identified for a wide range of infectious and non-infectious diseases, particularly for the identification and monitoring of seasonal trends in illness. Indicators used to monitor infectious diseases were reported in all 15 countries/territories. Respiratory infection indicators were described in all 15 countries/territories (influenza surveillance was specifically mentioned in 13/15 countries/territories) and infectious gastrointestinal illness indicators were described in 13/15 countries/territories (Table 2).

**Table 2:** Summary of emergency department syndromic surveillance systems (EDSyS) included in the review, by country/territory, with source and format of information used to define syndromic indicators and of areas of public health surveillance supported the EDSyS

Country/territory	Syndromic indicator		Infectious diseases			Extreme weather		Other non-infectious		
	Source^a^	Format	Respiratory	Influenza	Gastrointestinal	Heat	Cold	Injury/trauma	alcohol	drug
Albania	diagnosis	coded	**✓**	**✓**	**✓**	–	–	–	–	–
Australia	diagnosis	coded	**✓**	**✓**	**✓**	**✓**	**✓**	**✓**	**✓**	**✓**
Canada	chief complaint	text	**✓**	**✓**	**✓**	**✓**	**✓**	**✓**	–	–
China	chief complaint	coded	**✓**	**✓**	–	–	–	–	–	–
France	diagnosis	coded	**✓**	**✓**	**✓**	**✓**	**✓**	**✓**	**✓**	–
Greece	chief complaint	pick list	**✓**	–	**✓**	–	–	–	–	–
Italy	chief complaint	text/coded	**✓**	**✓**	**✓**	–	–	–	–	–
Jamaica	“daily analysed data”		**✓**	–	**✓**	**✓**	–	–	–	–
Republic of Korea	diagnosis	coded	**✓**	**✓**	**✓**	–	–	–	–	–
New Zealand	diagnosis	coded	**✓**	**✓**	–	–	–	–	–	–
Singapore	unknown	coded	**✓**	**✓**	**✓**	–	–	–	–	–
Spain	chief complaint	coded	**✓**	**✓**	**✓**	–	–	–	–	–
Taiwan	chief complaint	text/ coded	**✓**	**✓**	**✓**	–	–	–	–	–
UK^b^	diagnosis	coded	**✓**	**✓**	**✓**	**✓**	**✓**	–	**✓**	–
USA	chief complaint	text	**✓**	**✓**	**✓**	**✓**	**✓**	**✓**	**✓**	**✓**

^a^ EDSyS may collect more than one data item for syndromic indicators, but each reported a primary field used as standard

^b^ UK: England & Northern Ireland **✓** relevant EDSyS indicators identified - no relevant EDSyS indicators identified

The development and application of non-infectious syndromic indicators was also reported, particularly for the impact of weather events (storms/hurricanes –chiefly in the USA and hot (6/15) and cold (5/15) weather); injury surveillance (4/15); impact of alcohol (4/15) and drugs (2/15)) (Table 2).

### Indicator mapping

For methods used in the mapping of ED data to syndromic indicators there was an apparent divergence between EDSyS systems based in (or using a surveillance tool developed in) North America and other countries. Non-North American systems primarily use coded diagnosis information (most commonly International Classification of Diseases (ICD) and Snomed code sets; Table 2). Over the time period included in this review there was increasing provision of coding lists adding detail of which diagnoses were selected for the various indicators used in EDSyS systems using coded diagnostic information.

Conversely, EDSyS systems and surveillance tool solutions developed in North America primarily used chief complaints or triage/signs/symptoms collected as text, which is then mapped to syndromic indicators. These fields are cited as being available more closely to real-time than diagnostic coded information, which is often also collected at a later point in time (in ICD code format) [90–96].

The complexity of indicator recognition in a (free) text-based system is much greater than in a coded system. Text-based chief complaint EDSyS systems regularly provided case definitions [97–101], keywords used (including negations) [99, 102–104], or simply described the use of an algorithm (either bespoke [54], or ‘CoCo’ [67, 76]). Although free text chief complaint data was the primary source of information for the identification of a syndromic indicator, diagnosis data was collected where available and also used to supplement indicator development [90, 104].

### Information for public health action

All systems reported the use of EDSyS data to identify and monitor incidents of public health importance. Effective communication is necessary between those administering the surveillance and those responsible for public health action in order for the EDSyS system to enable swift public health action.

EDSyS systems which collected data at a patient level (ie not aggregated) were mostly designed and run to collect patient identifiable information (defined here as patient name, date of birth, full postal/zip code or any ID number not unique to the EDSyS system). The use of patient identifiable information (PII) supported local health protection functions through the identification of individual cases or contacts of infectious disease (e.g. gastrointestinal outbreaks [105–107], measles contact tracing [108], TB case follow-up [109]). One EDSyS was reported to have the facility to include PII if required, however the use of PII was not routine [44].

A small number of systems, working at both national (France [110] and UK [111]) and sub-national levels (Canada [67], Australia [112] and USA [66, 112, 113]) were specifically stated to be restricted to the collection of non PII data only. The methods for this anonymisation included the use of patient age in years (rather than date of birth) and partial postal/zip codes. The surveillance outputs from these EDSyS systems were reportedly communicated to public health protection colleagues, similar to the non-anonymised systems, although individuals could not be directly identified and followed up from this data source alone.

The methods used to communicate the findings of EDSyS to local public health colleagues ranged from the provision of summary reports [114] to the sharing of line listings of cases [93, 115]. In some instances, direct online access to the ED surveillance database or bespoke surveillance dashboards was described as being available to those working in public health [116–118]. The EDSyS systems in France and the UK reported the regular publication of national surveillance findings on publicly available platforms [111, 119].

### Cross-system working

EDSyS systems have been developed and implemented separately in multiple locations, however, collaborations between systems for public health risk assessment and investigation purposes have been reported. Within the USA, cross-system collaboration crossing multiple health/government jurisdictions was identified for particular events [120, 121], for increased coverage across sub-national borders [122, 123] and in response to an outbreak/incident [45, 124].

These collaborations developed further over time with the move to a single National Syndromic Surveillance Program (NSSP) across the USA (building upon the earlier DiSTRiBute and BioSense systems) [125]. The consolidation into NSSP has aided in collaborative working across larger areas of the USA as well as introducing EDSyS where it had not previously been available [126]. This collaboration demonstrated the evolution of a locally developed EDSyS systems into a national network.

Examples of public health process research (rather than data combining/sharing) were found across EDSyS in Canada [127–129]. Collaborative working across international borders was identified less often. The RODS tool had been reported to be used for EDSyS in Canada, Taiwan and USA, however, outside of the USA no international cross-border use of the tool was identified [130].

A single report of an international cross-EDSyS system collaboration was identified where the impact of poor air quality was examined using EDSyS data from EDSyS systems in France and the UK [131]. One other instance of potential cross border working was identified, however it relied on a comparison with a bespoke ED data collection, rather than a second syndromic surveillance system [132].

### Evolution

The evolution of EDSyS was a recurring theme of the studies identified. Expanding coverage, improved data quality/completeness and more real-time surveillance have become the norm. Several of the earliest ED systems utilised a ‘drop in’ surveillance format, requiring relatively labour-intensive manual data collection processes, before the manual transfer of information to a central surveillance point [30, 45].

Developments in technology have facilitated improvements in data collection in EDs and accessibility of the data from the ED clinical patient record. These changes have provided opportunities for EDSyS, allowing extraction of data from EDs with secure and automated processes transferring data to EDSyS databases. These processes in turn require no extra work from data providers. The frequency of collection in these systems varies from ‘near real-time’ (i.e. the collation and transfer of data on a daily basis [133–135] or more frequently [63, 65]), to truly ‘real-time’ (i.e. data available as entered in the ED system, or very soon after) [67, 77, 136].

Furthermore, the availability of ED data has further improved as the working practice in the ED has changed to collect electronic clinical information. This change has removed the need to wait for a data entry clerk to enter billing information or even paper-based diagnosis records several days later. These factors increase the potential for diagnosis information to be made available, along with other details such as clinical measurements carried out in the ED.

## Discussion

With the relatively common provision of ED services globally it is therefore unsurprising that EDSyS systems were identified in 15 countries and territories, on four different continents. The earliest EDSyS systems identified in this review were created in 1999 and are some of the first examples of syndromic surveillance in general. However, the references describing these systems (or their use) were not published until several years later. The earliest EDSyS paper identified was published during September 2002 [53], 2 weeks before the first ISDS conference (which was the US National Syndromic Surveillance Conference at that time) [14].

Historically the threat from bioterrorism provided much impetus as well as funding for the early development of syndromic surveillance, and in particular EDSyS systems [54, 88, 89, 137, 138]. The bioterrorism threat has also influenced the need for more timely public health reporting and action, necessitating rapid surveillance activities. Though some EDSyS systems were identified to collect truly real-time data, the majority of EDSyS activities appear to have settled to a daily rhythm of analysis and reporting. The daily time frame is in most instances both necessary and appropriate (simplifying the transfer and storage of data by allowing time for records to be completed during the patient journey through the ED and sent at a time when the local network is less busy, rather than continually updated/refreshing/transmitting) whilst also enabling provision of easily understood and actionable information in a suitable timeframe for action by public health authorities, which do not generally work on a minute-by minute basis.

EDSyS has been shown to be an effective form of public health surveillance, providing information for action (or even reassurance of no public health impact) across a wide range of situations, both infectious and non-infectious conditions, during seasonal and sporadic events. Although initially largely focused on infectious diseases (particularly influenza) EDSyS has developed to encompass many of the different types of conditions seen and treated in EDs, providing information for public health action. This valuable source of data augments laboratory surveillance of infectious diseases (providing information more quickly than laboratory systems and on those conditions for which a confirmatory test may not be carried out) and extends the ability of public health to identify and respond to non-infectious diseases in a timelier manner than would be possible without EDSyS.

An important feature of the early examples of EDSyS was rapid system establishment to provide valuable public health information for action in preparation for known mass gatherings and/or in response to an outbreak/unanticipated events. These early versions provided the first evidence of the value of EDSyS, whilst highlighting the limitations in terms of the workload and sustainability, particularly of drop-in systems. Technological and working practice developments within EDs, which have occurred for patient care purposes (particularly the immediate collection and storage of electronic patient records), have enabled developments in the automation of secure data collection and transfer for EDSyS purposes. The greater opportunity for secure automated data collection has made EDSyS data collection easier and more sustainable.

As a result, EDSyS systems are developing rapidly and largely in the same direction: utilising electronic patient ED records which are completed immediately and can be made available for public health surveillance rapidly. The observed dichotomy between systems utilising either chief complaint or coded diagnosis data may become less distinct in future. EDSyS systems may base their indicators primarily on either diagnosis codes or chief complaints, however, in practice they generally collect both data fields when they are available. With coded diagnosis data being made available more quickly and methods for working with text based chief complaint data becoming more mainstream, the use of both chief complaint and coded diagnosis data to group clinical encounters/episodes into syndromic indicators is likely to become standard. Additional detail may also be added as appropriate, such as clinical measurements e.g. body temperature.

It is important to acknowledge that while EDSyS systems comprise some of the earliest examples of syndromic surveillance systems, there are examples of other morbidity sentinel surveillance networks that were operational decades before EDSyS. Sentinel surveillance systems such as the Royal College of General Practitioners Weekly Returns Service (England) and the French ‘Réseau Sentinelles’ physician network have been collecting weekly returns of community-based morbidity data using semi-automated methods since 1966 and 1984 respectively [139, 140].

### Strengths and limitations of this review

Through the identification and interrogation of both journal articles and abstracts/papers from syndromic surveillance themed conferences, we were able to identify a large number of EDSyS systems, in more countries/territories than would have been possible from journal articles alone. The exclusion of non-English language publications may still have limited the findings of this review. Other novel descriptions of EDSyS systems, such as websites and reports are also likely to have added further detail, though may not be searchable in a systematic manner.

As the terminology for healthcare provision is not globally standardised this review relied on the identification of studies including the term ‘emergency’ (while allowing for global variation with the addition of room/department/care) or an indication of data collected during unscheduled emergency hospital care (such as triage) in the title and/or abstract. In the absence of these terms any other EDSyS that is described as ‘hospital’ based syndromic surveillance systems will have been excluded. Furthermore, the description of EDSyS in the literature is occasionally obscured by the use of names of syndromic surveillance systems and tools in titles and abstracts, rather than explicitly describing the use of data from an ED source. These difficulties may be due to the surveillance system being reported collecting data from a range of sources. Several syndromic surveillance systems collect data from multiple data sources (e.g. ambulance call outs, poison centre calls, and/or over the counter sales), with analysis and interpretation on a whole system basis, rather than a single data source. We are aware that a number of references excluded during the identification phase of this review were indeed related to EDSyS, but did not include any term related to the ED in the title or abstract, instead relying on the reader being familiar with the system name (e.g. ESSENCE or RODS both of which were described elsewhere in references used in this review).

The level of information available in conference abstracts in particular was minimal in some cases, providing little detail other than an EDSyS system existed. These references instead focused on a research question (such as a natural language processing algorithm, or a statistical technique). Discussion of research in both conferences and the published literature is important, however the day-to-day working and the value added to public health by EDSyS was less obvious. The inclusion of multiple information sources for each EDSyS when found (both research papers and conference abstracts), allowed the available information to be pieced together, filling in gaps where possible.

In those countries with large numbers of standalone EDSyS systems, e.g. Canada and USA, there is potential for this review to have incorrectly estimated the number of EDSyS systems, as not all have been described individually. The evolution of systems over time with occasional overlap/merging of once separate systems, or addition of new national surveillance layers above what may still remain as stand-alone systems locally is, however an encouraging sign that EDSyS continues to be used and developed. Geographical (and population) coverage is increasing, aiding in both the developments of systems themselves, but importantly increasing the potential to achieve the primary aim of providing information for public health action.

Finally, it is inevitable that between the execution of this review and the peer review publishing of results there will have been further developments or significant events in the field that the review does not capture, for example the COVID-19 pandemic. Whilst this can’t be avoided, we acknowledge that the EDSyS systems included in this systematic review will not capture all systems in operation at the time of publication. Reviews of this kind require continual updating to remain timely and representative.

### Future work and developments

This review provides a pragmatic exploration and description of international EDSyS, giving some insight into where, and how, it has been used and how systems have evolved over time. A previous review focussed solely on the use of EDSyS for influenza surveillance [10]. Similar detailed reviews may be useful for the description of other syndromic indicators or even the statistical methods in use or even those proposed for or discounted from use in future.

Increased sharing of indicator detail (diagnosis coding lists/algorithms for free text processing) will enable syndromic surveillance systems to learn from each other. Further developments in the standardisation of, and increased breadth of, information available from electronic patient records and real-time entry of data into the ED patient record are allowing for additional, more granular detail to be made available in (near) real-time for surveillance purposes. The collection of patient observation details, particularly temperature, has been discussed for the more reliable identification of patients attending ED with a clinical fever (rather than self-reported) [141]. Future exploration into the use of combinations of data fields from the ED patient care record (e.g. diagnosis/chief complaint/tests and measurements) for the identification of syndromic indicators should be carried out to utilise and expand on the experience gained through the past 20 years of EDSyS globally.

Artificial intelligence (AI) and deep machine learning are exciting areas of development within syndromic surveillance. These methods have the potential to improve analysis tools, detection algorithms and syndromic surveillance activities in general. However, because of the relative recent advent of these technologies they have not been included in this systematic review. A further review of the application of AI and deep machine learning in syndromic surveillance would be an interesting and relevant addition to this field. Furthermore, the timing of this review has precluded the COVID-19 pandemic, which has further highlighted the importance of EDSyS [142, 143]. It will be important to undertake a future systematic review of EDSyS in the aftermath of COVID-19 to assess changes to EDSyS globally and how systems were used in response to the pandemic [144].

The monitoring of syndromic indicators of public health importance is effective and, in some situations, provides the only real-time method for monitoring rapidly evolving events. The identification of similarities between EDSyS systems presents opportunities for harmonisation and collaboration in future. The USA has developed NSSP [125], there has been an investigation of cross-border working in Europe with the Triple S project [145] and the first examples of multi country, multi EDSyS analysis in France and the UK [146, 147].

Infectious and of non-infectious disease events of public health importance do not respect geopolitical borders. Additionally, patients may cross these borders when seeking/receiving health care. Countries with linked and unified health information systems have a major advantage for EDSyS system development, but unified systems are rarely applied across borders. Therefore, cross-border cooperation is a vital and necessary development for EDSyS and wider syndromic surveillance. International cooperation and collaborations to oversee a coordinated syndromic surveillance approach would strengthen public health surveillance. The ISDS developed such a model providing a much-needed international forum for sharing and discussing ED syndromic surveillance, as evidenced by the number of EDSyS identified from conference abstracts (including several not identifiable elsewhere in the literature). However, during 2019 a loss of funding resulted in the dissolution of ISDS: the field of syndromic surveillance has since missed the leadership of ISDS, particularly during the COVID-19 pandemic. The ‘Triple-S’ program also sought a programme of syndromic surveillance standardisation across Europe, however, without ongoing funding this initiative was not sustained. However, the trans-European system EUROMOMO demonstrates a positive example of sustained cross-border surveillance of mortality data across Europe illustrating the benefits of such networks [148]. The field of syndromic surveillance would benefit again from such international collaborative programmes.

## Conclusions

This systematic review included 559 studies describing 115 EDSyS systems across 15 countries/territories. EDSyS was found to provide a valuable tool for the identification and monitoring of trends in those seeking care within the ED setting, for both infectious and non-infectious disease. Although individual EDSyS systems have been developed independently across various geographies in multiple countries/territories, many similarities were identified with opportunities for cross-system learning. There is potential for further system developments, collaborative working and even harmonisation between systems in future. This review provides the first description of EDSyS globally and reveals how ED clinical system evolution has provided the potential for future growth of EDSyS, both geographically and in the development and refinement of surveillance tools for new and existing areas of public health concern.

## Supplementary Information

**Additional file 1**. Emergency Department syndromic surveillance (EDSyS) systems identified, number of EDs included, dates of system start, most recent data and publication, by country and geographical coverage, with full reference details. Full list of all 559 studies identified for inclusion in this review, separated into journal articles and conference abstracts. References are grouped by EDSyS system, detailing the country/territory, state/province, county/region and city/hospital included in each. The number of EDs in each EDSyS system, the year the EDSyS system started, the year of latest data and year of last publication identified are described.

## Data Availability

Data sharing is not applicable to this article, however the full list of 558 studies included in this review (presented by individual system, detailing the geography, coverage, timing and reference type for each) is given in Additional File 1.

## Abbreviations

AI: Artificial Intelligence
ED: Emergency Department
EDSyS: ED syndromic surveillance
ESSENCE: Electronic surveillance system for the early notification of community-based epidemics
ICD: International classification of disease
ISDS: International society for disease surveillance
NSSP: National syndromic surveillance program
PII: Patient identifiable information
PRISMA: Preferred reporting items for systematic reviews and meta-analyses
RAMMIE: Rising activity, multi-level mixed effects, indicator emphasis
RODS: Real-time outbreak and disease surveillance
UK: United Kingdom
USA: United States of America
