# Supplementary material for: Emergency department syndromic surveillance systems: a systematic review
Source: BMC Public Health. 2020 Dec 9;20:1891. doi: 10.1186/s12889-020-09949-y (PMC7724621; doi:10.1186/s12889-020-09949-y)
Supplement: Supplementary file 1 — Additional file 1. Emergency Department syndromic surveillance (EDSyS) systems identified, number of EDs included, dates of system start, most recent data and publication, by country and geographical coverage, with full reference details. Full list of all 559 studies identified for inclusion in this review, separated into journal articles and conference abstracts. References are grouped by EDSyS system, detailing the country/territory, state/province, county/region and city/hospital included in each. The number of EDs in each EDSyS system, the year the EDSyS system started, the year of latest data and year of last publication identified are described. [file 12889_2020_9949_MOESM1_ESM.docx]

**Additional file 1:** Emergency Department syndromic surveillance (EDSyS) systems identified, number of EDs included, dates of system start, most recent data and publication, by country and geographical coverage, with full reference details

| **Country/ territory** | **State/ Province** | **County(ies)/ region** | **City/ Hospital** | **Coverage level** | **Max EDs included** | **Start year (estimated/ earliest data reported)** | **Latest data** | **Latest publication** | **Journal article references** | **Conference abstract references** |
| --- | --- | --- | --- | --- | --- | --- | --- | --- | --- | --- |
| Albania | - | - | - | National | Hospitals in all 36 districts | (2013) | - | 2014 | (1) |  |
| Australia | New South Wales | - | - | State | 68 hospitals, 85% of ED activity | 2003 | 2014 | 2016 | (2-17) | (18, 19) |
|  | Victoria | - | Melbourne | Hospital(s) | 2 EDs | 2005 | 2009 | 2011 | (20) |  |
|  | Victoria | - | Melbourne | Hospital | 1 ED | 2006 | 2008 | 2010 | (21) |  |
| Canada | Alberta | Edmonton | - | Public Health Unit | - | (2007) | 2011 | 2013 |  | (22, 23) |
|  | Manitoba | - | Winnipeg | City | 7 EDs | (2006) | 2011 | 2014 | (24) | (25, 26) |
|  | Ontario | - | - | Province | 132 hospitals, >80% of ED visits in Ontario | 2004 | 2016 | 2017 | (27-36) | (37-48) |
|  |  | 11-15 Public Health Units | - | Public Health Unit | - | (2009) | 2015 | 2017 | (36, 49) | (48) |
|  |  |  | Toronto | City | - | (2002) | 2002 | 2004 |  | (47) |
|  | Quebec | - | Montreal | City | 5 hospitals, 28% ED visits | (2006) | 2001 | 2015 | (50) |  |
| China | - | - | Beijing | City | 2 major hospitals | (2004) | 2004 | 2006 |  | (51) |
|  | - | - | Wuxu | City | 2-3 hospitals | (2004) | 2009 | 2011 |  | (52) |
| France | - | - | - | National | >650 EDs, 88% of all ED visits | 2004 | 2016 | 2017 | (53-59) | (60-84) |
| Greece | - | - | Athens + Olympic host cities | Multi city | 17 hospitals | 2002 | 2003 | 2003 |  | (85, 86) |
| Italy | - | - | Genoa | City | 72% of ED visits | 2007 | 2013 | 2015 | (87-90) |  |
|  | - | Lazio | - | Region | 36/61 EDs | 2000 | 2004 | 2009 | (91) |  |
| Jamaica | - | - | - | National | - | 2007 | 2007 | 2007 |  | (92) |
| Republic of Korea | - | - | - | National | 125 EDs | 2002 | 2009 | 2010 | (93, 94) | (95) |
| New Zealand | - | - | Wellington | Hospital | 1 ED | 2008 | 2008 | 2009 | (96) |  |
| Singapore | - | - | - | National | 7 hospitals | (2013) | 2013 | 2013 |  | (97) |
| Spain | - | - | Santander | Hospital | 1 ED | (2010) | 2012 | 2014 | (98, 99) |  |
| Taiwan | - | - | - | National | 170 hospitals: 85% of all visits (earlier study included 189 hospitals but no % value) | 2003 | 2012 | 2013 | (27, 100, 101) | (102, 103) |
|  | - | - | Taipei | City | 5 Hospitals | (2005) | 2008 | 2008 |  | (104-106) |
| UK | - | - | - | National | 36 EDS | 2010 | 2015 | 2017 | (107-121) | (84, 122, 123) |
| USA  USA  USA  USA  USA  USA  USA  USA | DiSTRIBuTE | - | - | National | >50 state/ local health dept ED syndromic systems | 2005 | 2009 | 2012 | (124) | (125-133) |
|  | Biosense/  NSSP |  |  | National | 4,000 hospitals, 55% ED visits | 2003 | 2016 | 2017 | (134-141) | (142-173) |
|  | Military | - | - | National | >300 military treatment facilities worldwide | (1999) | 2016 | 2010 | (174) | (175-178) |
|  | Multistate (not named) |  |  | MultiState | - | (2003) | 2003 | 2008 | (27) |  |
|  | Arizona | - | - | State | 15 EDs | 2001 | 2001 | 2004 | (179) | (180) |
|  |  | Maricopa | - | County | 11 hospitals | 2001 | 2004 | 2005 |  | (181, 182) |
|  | California | Los Angeles | - | County | >65% of ED patients | 2003 | 2016 | 2017 | (183) | (184-195) |
|  |  | Santa Clara | - | County | 12 EDs | 2001 | 2002 | 2002 |  | (196) |
|  |  | San Diego | - | County | 16 hospitals - 86% ED visits | 1999 | 2007 | 2013 | (197) | (198-202) |
|  | Colorado | - | - | State | 17 EDs | (2015) | 2015 | 2015 |  | (203) |
|  |  | - | Denver | City | 9 EDs | 2003 | 2003 | 2003 |  | (204) |
|  | Connecticut | - | - | State | 21/32 EDs | 2004 | 2011 | 2012 |  | (205-212) |
|  | Florida | - | - | State | 231 of 240 EDs, 96% of EDs | 2006 | 2016 | 2016 | (213, 214) | (215-230) |
|  |  | Broward | - | County | - | 2005 | 2007 | 2007 |  | (231, 232) |
|  |  | Cook | - | County | - | 2007 | 2007 | 2007 |  | (233) |
|  |  | Duval | - | County | 8/10 EDs | 2007 | 2008 | 2011 |  | (234-239) |
|  |  | Hillsborough, Pinellas and Collier | - | multi County | 9 EDs | 2001 | 2001 | 2006 |  | (240, 241) |
|  |  | Miami-Dade | - | County | 17 largest of 23 EDs | 2005 | 2010 | 2011 |  | (233, 242-257) |
|  | Georgia | - | - | State | 112 EDs | 2005 | 2016 | 2016 |  | (258-267) |
|  | Illinois | - | - | State | - | (2012) | 2013 | 2013 |  | (268) |
|  |  | Champaign | - | County | - | (2007) | 2011 | 2011 |  | (269) |
|  |  | Cook | - | County | 45 EDs | (2012) | 2015 | 2017 | (270) | (271-273) |
|  |  | - | Chicago | City | 1 ED | (2012) | 2012 | 2012 |  | (274) |
|  | Indiana | - | - | State | 110 hospitals, 90% of ED visits | 2004 | 2010 | 2014 | (275, 276) | (233, 277-285) |
|  |  | Marion | - | County | 14 EDs | 2007 | 2011 | 2011 |  | (233, 286) |
|  | Kentucky |  | Louisville | City | 9 EDs | 2002 | 2002 | 2005 | (287) | (288) |
|  | Louisiana | - | - | State | 11 EDs ( in 2013) | 2005 | 2016 | 2016 |  | (289-294) |
|  | Massachusetts | - | Boston | City | 10/10 EDs | 2004 | 2011 | 2012 | (295, 296) | (239, 297-309) |
|  | Maryland | - | - | State | 47 EDs 100% of EDs | 2005 | 2012 | 2013 |  | (310-320) |
|  |  | - | Baltimore | City | 11/11 EDs | - | - | 2002 |  | (321) |
|  | Maine | - | - | State | 24 of 37 EDs | 2007 | 2012 | 2012 |  | (322) |
|  |  | - | Maine Medical Centre, Portland | Hospital | 1 ED | 2002 | 2004 | 2004 |  | (323) |
|  | Michigan | - | - | State |  | 2006 | 2014 | 2014 |  | (324-326) |
|  | Minnesota | - | Childrens hospital - Roseville | Hospital | - | (2003) | 2003 | 2004 |  | (327) |
|  |  | - | Hennepin County Medical Center, Minneapolis | Hospital | 1 ED | (2001) | 2003 | 2003 |  | (328) |
|  | Missouri | - | - | State | 84 EDs, 90% visits | 2001 | 2013 | 2013 |  | (329-335) |
|  | Montana | - | - | State | - | - | - | 2008 |  | (336) |
|  | North Carolina | - | - | State | 122 EDs, around 4.8 million visits per year | 1999 | 2015 | 2017 | (337-341) | (342-374) |
|  |  | Military | - | State | 1 ED | 2002 | 2007 | 2007 |  | (375) |
|  | North Dakota | - | - | State | 1 ED | (2005) | 2008 | 2008 |  | (376, 377) |
|  | Nebraska | - | - | State | 32 facilities | (2011) | 2016 | 2016 |  | (378-380) |
|  | New Hampshire | - | - | State | all acute hospitals by 2010 | 2001 | 2015 | 2017 | (381) | (382-386) |
|  | New Jersey | - | - | State | 79/80 EDs | 2001 | 2016 | 2017 | (387, 388) | (389-399) |
|  |  | Bergen | - | County | 6/6 EDs | 2001 | 2004 | 2004 |  | (400, 401) |
|  | New Mexico | - | Albuquerque | City | 2 EDs in 1 hospital | 2002 | 2003 | 2005 | (402) |  |
|  | New York | - | - | State | 140 EDs, 4 million visits per year | (2004) | 2012 | 2017 | (403) | (404-411) |
|  |  | Westchester | - | County | 7/13 EDs, approx 80% visits | 2003 | 2003 | 2003 |  | (412, 413) |
|  |  | - | New York | City | 51 EDs (98% of ED visits) | 2001 | 2003 | 2017 | (414-433) | (239, 434-475) |
|  | Ohio | - | - | State | 96% of EDs | 2003 | 2012 | 2012 |  | (285, 476-481) |
|  |  | - | Akron | City | 3 EDs | 2003 | 2003 | 2003 |  | (204) |
|  | Oklahoma | Tulsa | - | County | 7 hospitals | 2002 | 2006 | 2007 |  | (482) |
|  | Oregon | - | - | State | 60/60 EDs | (2011) | 2016 | 2017 | (483) | (484-488) |
|  | Pennsylvania | SW state |  | Regional | - | 1999 | 2002 | 2004 | (489) |  |
|  |  | Allegheny | - | County | 7 EDs | 2009 | 2009 | 2010 |  | (490) |
|  |  | - | Philadelphia | City | - | (2006) | 2011 | 2017 | (491) |  |
|  | Rhode Island | - | - | State | all 11 EDs | (2006) | 2013 | 2013 |  | (492-494) |
|  | South Carolina | - | - | State | 22 hospitals | (2008) | 2012 | 2013 |  | (495-499) |
|  |  | - | Greenville hospital system | Hospital(s) | a level I trauma center... plus 3 smaller outlying ERs | 2003 | 2007 | 2007 |  | (500, 501) |
|  | Tennessee | Knoxville | - | County | 3 EDs | 2002 | 2002 | 2005 | (502) |  |
|  | Texas | - | Dallas | City | - | (2012) | 2012 | 2013 | (503) |  |
|  |  | - | Fort Worth | City | 2 hospitals | 2003 | 2003 | 2003 |  | (204) |
|  |  | Harris | - | County | 34 hospitals, 70% ER beds | 2004 | 2014 | 2014 |  | (504-509) |
|  |  | Travis | - | County | 14 hospitals | 2010 | 2011 | 2011 |  | (510) |
|  |  | Health service region 8 | - | Region | 3 hospitals | 2006 | 2006 | 2007 |  | (511) |
|  | Utah | Salt Lake | - | County | 15 sites (EDs + Urgent Care Centres) | (2009) | 2009 | 2010 |  | (512) |
|  | Virginia | - | - | State | 82 EDs | (2008) | 2016 | 2016 |  | (320, 513-517) |
|  |  | Tidewater or Hampton Roads region | - | Region | 7 EDs | (2002) | 2002 | 2003 |  | (518) |
|  | Washington | King | - | County | 19/20 EDs | 1999 | 2013 | 2014 | (519-521) | (239, 522-532) |
|  |  | Kitsap, heath district | - | Multi County | 3 EDs | (2004) | 2006 | 2007 |  | (532-534) |
|  |  | Pierce | - | County | 5 EDs (approx 80% of ED records in county) | (2004) | 2005 | 2007 |  | (532, 535) |
|  |  | Spokane | - | County | 4 major community hospitals | - | - | 2007 |  | (536) |
|  | Wisconsin | - | - | State | 14 acute-care hospitals in 6 counties | 2008 | 2009 | 2009 |  | (537) |
|  |  | - | Milwaukee | City | 11 departments | 2002 | 2003 | 2004 | (538, 539) | (204, 540) |
|  |  | - | University of Wisconsin Hospital | Hospital | 1 ED | (2007) | 2009 | 2011 | (541) | (542, 543) |
|  | Washington DC | - | - | DC | 9 hospitals | 2001 | 2016 | 2017 | (544, 545) | (546-548) |
|  |  | Capital region - including Virginia and Maryland | - | Region | 30% of acute care hospitals in state, 90% in national capital region | 2001 | 2009 | 2009 | (549, 550) | (551-553) |
|  |  | - | DC university hospitals | Hospital(s) | 2 University hospitals | (2009) | 2009 | 2010 |  | (554) |
| Unknown | Unknown | Unknown | Unknown | Unknown | - | (2013) | 2016 | 2016 |  | (555, 556) |

Reviews describing multiple systems (all included in the table above): (557-559)

**References:**

1. Simaku A, Ulqinaku D, Hatibi I, Robo A, Kakarriqi E, Bino S. Syndromic surveillance for detection of influenza in Albania. Albanian Journal of Agricultural Sciences. 2014;13(1):16-21.

2. Polkinghorne BG, Muscatello DJ, MacIntyre CR, Lawrence GL, Middleton PM, Torvaldsen S. Relationship between the population incidence of febrile convulsions in young children in Sydney, Australia and seasonal epidemics of influenza and respiratory syncytial virus, 2003-2010: a time series analysis. BMC Infectious Diseases. 2011;11(291).

3. Hope KG, Merritt TD, Durrheim DN, Massey PD, Kohlhagen JK, Todd KW, et al. Evaluating the utility of Emergency Department syndromic surveillance for a regional public health service. Communicable Diseases Intelligence. 2010;34(3):310-8.

4. Schindeler SK, Muscatello DJ, Ferson MJ, Rogers KD, Grant P, Churches T. Evaluation of alternative respiratory syndromes for specific syndromic surveillance of influenza and respiratory syncytial virus: a time series analysis. BMC Infectious Diseases. 2009;9(190).

5. Zheng W, Aitken R, Muscatello DJ, Churches T. Potential for early warning of viral influenza activity in the community by monitoring clinical diagnoses of influenza in hospital emergency departments. BMC Public Health. 2007;7(250).

6. Turner RM, Muscatello DJ, Zheng W, Willmore A, Arendts G. An outbreak of cardiovascular syndromes requiring urgent medical treatment and its association with environmental factors: an ecological study. Environmental Health. 2007;6(37).

7. Muscatello DJ, Churches T, Kaldor J, Zheng W, Chiu C, Correll P, et al. An automated, broad-based, near real-time public health surveillance system using presentations to hospital Emergency Departments in New South Wales, Australia. BMC Public Health. 2005;5(141).

8. Dinh MM, Kastelein C, Bein KJ, Green TC, Bautovich T, Ivers R. Use of a syndromic surveillance system to describe the trend in cycling-related presentations to emergency departments in Sydney. Emergency Medicine Australasia. 2015;27(4):343-7.

9. Cashmore AW, Muscatello DJ, Merrifield A, Spokes P, Macartney K, Jalaludin BB. Relationship between the population incidence of pertussis in children in New South Wales, Australia and emergency department visits with cough: a time series analysis. BMC Medical Informatics and Decision Making. 2013;13:40.

10. Hope K, Merritt T, Eastwood K, Main K, Durrheim DN, Muscatello D, et al. The public health value of emergency department syndromic surveillance following a natural disaster. Communicable Diseases Intelligence. 2008;32(1):92-4.

11. Whitlam G, Dinh M, Rodgers C, Muscatello DJ, McGuire R, Ryan T, et al. Diagnosis-based emergency department alcohol harm surveillance: What can it tell us about acute alcohol harms at the population level? Drug and Alcohol Review. 2016;35(6):693-701.

12. Liljeqvist HTG, Muscatello D, Sara G, Dinh M, Lawrence GL. Accuracy of automatic syndromic classification of coded emergency department diagnoses in identifying mental health-related presentations for public health surveillance. BMC Medical Informatics and Decision Making. 2014;14:84.

13. New South Wales public health networ. Progression and impact of the first winter wave of the 2009 pandemic H1N1 influenza in New South Wales, Australia. Euro surveillance. 2009;14(42).

14. Cumming G, Khatami A, McMullan BJ, Musto J, Leung K, Nguyen O, et al. Parechovirus genotype 3 outbreak among infants, New South Wales, Australia, 2013–2014. Emerging Infectious Diseases. 2015;21(7):1144-52.

15. Schaffer A, Muscatello D, Broome R, Corbett S, Smith W. Emergency department visits, ambulance calls, and mortality associated with an exceptional heat wave in Sydney, Australia, 2011: A time-series analysis. Environmental Health: A Global Access Science Source. 2012;11(1).

16. Sparks R, Carter C, Graham P, Muscatello D, Churches T, Kaldor J, et al. Understanding sources of variation in syndromic surveillance for early warning of natural or intentional disease outbreaks. IIE Transactions (Institute of Industrial Engineers). 2010;42(9):613-31.

17. Hope K, Durrheim DN, Muscatello D, Merritt T, Zheng W, Massey P, et al. Identifying pneumonia outbreaks of public health importance: Can emergency department data aissist in earlier identification? Australian and New Zealand Journal of Public Health. 2008;32(4):361-3.

18. Muscatello DJ, Churches T, Kaldor J, Zheng W, Chiu C, Correll P, et al. Emergency department surveillance for the 2003 Rugby World Cup -- New South Wales, Australia.Third annual Syndromic Surveillance Conference. Boston, Massachusetts. November 3-4, 2004. MMWR: Morbidity & Mortality Weekly Report; Atlanta, Georgia 2005;54:196.

19. Zheng W, Muscatello DJ, Turner R, Neville L. The Potential of Syndromic Surveillance of Gastrointestinal Illness in the Community for Early Warning of Institutional Gastroenteritis Outbreaks. Advances in Disease Surveillance 2006;1:77.

20. Moore K, Black J, Rowe S, Franklin L. Syndromic surveillance for influenza in two hospital emergency departments. Relationships between ICD-10 codes and notified cases, before and during a pandemic. BMC Public Health. 2011;11(338).

21. Ramsay VC, Simpson P, Hobbs L, Sinickas V. An evaluation of a hospital influenza surveillance program using Emergency Department coding and laboratory data. Healthcare Infection. 2010;15(2):49-55.

22. Fan S, Brown A, Honish L, Hughes T, Jaipaul J, Mashinter L, et al. Enhancing Public Health Surveillance by Using Multiple Data Sources for Syndromic Surveillance: The Alberta Real Time Syndromic Surveillance Net. Advances in Disease Surveillance 2007;4:161.

23. Sikora C, Fournier K, Usman H, Jacobs A, Wicentowich B, Talbot J. Rapid Measles Exposure Assessment in an Urban Emergency Department Using a Syndromic Surveillance System. Online Journal of Public Health Informatics 2014; Vol 6, No 12014;6(1):e161.

24. Thompson LH, Malik MT, Gumel A, Strome T, Mahmud SM. Emergency department and 'Google flu trends' data as syndromic surveillance indicators for seasonal influenza. Epidemiology and Infection. 2014;142(11):2397-405.

25. McDonald L, Edge V, Aramini J, McDonald K. Evaluation of a Systematic Emergency Department Chief Complaint System for Near Real-Time Public Health Surveillance. Advances in Disease Surveillance 2007;2:206.

26. McDonald L, Guthrie G, Edge V, Aramini J. Detection Abilities of Several Commonly Used Algorithms as Determined by Simulation Analysis. Advances in Disease Surveillance 2007;2:205.

27. Krenzelok E, MacPherson E, Mrvos R. Disease surveillance and nonprescription medication sales can predict increases in poison exposure. Journal of Medical Toxicology. 2008;4(1):7-10.

28. Perry AG, Korenberg MJ, Hall GG, Moore KM. Modeling and syndromic surveillance for estimating weather-induced heat-related illness. Journal of Environmental and Public Health. 2011;2011.

29. van Dijk A, Dawson E, Moore KM, Belanger P. Risk Assessment During the Pan American and Parapan American Games, Toronto, 2015. Public Health Reports. 2017;132(1).

30. VanStone N, van Dijk A, Chisamore T, Mosley B, Hall G, Belanger P, et al. Characterizing the Effects of Extreme Cold Using Real-time Syndromic Surveillance, Ontario, Canada, 2010-2016. Public Health Reports. 2017;132:48S.

31. Hall GG, Perry AG, vanDijk A, Moore KM. Influenza assessment centres: a case study of pandemic preparedness to alleviate excess emergency department volume. Canadian Journal of Emergency Medicine. 2013;15(4):198-205.

32. Moore KM, Edgar BL, McGuinness D. Implementation of an automated, real-time public health surveillance system linking emergency departments and health units: rationale and methodology. Canadian Journal of Emergency Medicine. 2008;10(2):114-9.

33. van-Dijk A, Aramini J, Edge G, Moore KM. Real-time surveillance for respiratory disease outbreaks, Ontario, Canada. Emerging Infectious Diseases. 2009;15(5):799-801.

34. Hall G, Krahn T, Majury A, Van Dijk A, Evans G, Moore K, et al. Emergency department surveillance as a proxy for the prediction of circulating respiratory viral disease in Eastern Ontario. Canadian Journal of Infectious Diseases and Medical Microbiology. 2013;24(3):150-4.

35. Caudle JM, Van Dijk A, Rolland E, Moore KM. Telehealth ontario detection of gastrointestinal illness outbreaks. Canadian Journal of Public Health. 2009;100(4):253-7.

36. Rivera LA, Li Y, Savage RD, Crowcroft NS, Bolotin S, Rosella LC, et al. Evaluation of the ability of standardized supports to improve public health response to syndromic surveillance for respiratory diseases in Canada. BMC Public Health. 2017;17(1):199.

37. O’Connor KH, Moore KM, Edgar B, McGuinness D. Maximum Entropy Models in Chief Complaint Classification. Advances in Disease Surveillance 2007;2:23.

38. Moore K, Rimmer M, O’Connor K, McGuinness D, Edgar B. Emergency Syndromic Surveillance: Adapting Real-Time Outbreak and Disease Surveillance (RODS) for Public Health in Canada. Advances in Disease Surveillance 2006;1:50.

39. Moore K, Rimmer M, O’Connor K, McGuinness D, Edgar B. Integration of Hospital Admissions, Febrile Respiratory Illness Screening and Triage Acuity Score into an Emergency Department Syndromic Surveillance System. Advances in Disease Surveillance 2006;1:51.

40. Moore K, Rimmer M, O’Connor K, McGuinness D, Edgar B. Multi-sectored approach to evaluation of a syndromic surveillance system. Advances in Disease Surveillance 2006;1:52.

41. Moore KM, Edgar BL, McGuinness D, O’Connor K. Acute Care Alerting and Integrated Public Health Investigation and Response to Syndromic Surveillance Alerts: Example of a Local Food Borne Outbreak. Advances in Disease Surveillance 2007;2:113.

42. Moore KM, Edgar BL, McGuinness D, O’Connor K. Emergency Department Surveillance of Alcohol-related Violence and Injuries to Enable Event Monitoring and Management. Advances in Disease Surveillance 2007;2:162.

43. Moore KM, Edgar BL, McGuinness D, O’Connor K. Chart review of laboratory-confirmed Salmonella Enteritidis cases in a local food borne outbreak linked to mung bean sprouts. Advances in Disease Surveillance 2007;2:207.

44. Dijk Av, McPherson M, Sambol C, Moore K. Infection Watch Live - A real-time, geospatial mapping tool of hospital triage data for public consumption. Advances in Disease Surveillance 2008;5:194.

45. Moore KM, McGuinness D, Edgar BL, O'Connor K. Early Detection and Integrated Response to Seasonal Influenza. Advances in Disease Surveillance 2007;2:114.

46. Donovan TL, Moore KM, VanDenKerkhof L, McGuinness D, Edgar B. Absenteeism Among Employees in a Southeastern Ontario Hospital: A Novel Application of Syndromic Surveillance. Advances in Disease Surveillance 2007;2:152.

47. Bassil KL, Henry B, Rea E, Varia M, Cole D. Public Health Surveillance for World Youth Day -- Toronto, Canada, 2002. MMWR: Morbidity & Mortality Weekly Report 2005;54:183.

48. Rivera L, Savage R, Crowcroft N, Rosella L, Ye L, Bolotin S, et al. Characterizing Public Health Actions in Response to Syndromic Surveillance Alerts. Online Journal of Public Health Informatics 2016; Vol 8, No 12016;8(1):e34.

49. Chu A, Savage R, Whelan M, Rosella LC, Crowcroft NS, Willison D, et al. Assessing the Relative Timeliness of Ontario's Syndromic Surveillance Systems for Early Detection of the 2009 Influenza H1N1 Pandemic Waves. Canadian Journal of Public Health. 2013;104(4):340-7.

50. Savard N, Bédard L, Allard R, Buckeridge DL. Using age, triage score, and disposition data from emergency department electronic records to improve Influenza-like illness surveillance. Journal of the American Medical Informatics Association: JAMIA. 2015;22(3):688-96.

51. Zhang X, Ding Y, Chen Z, Schable C. Development of an Integrated Surveillance System for Beijing. Advances in Disease Surveillance 2007;2:127.

52. Qian YH, Su J, Shi P, He EQ, Shao J, Sun N, et al. Attempted early detection of influenza A (H1N1) pandemic with surveillance data of influenza-like illness and unexplained pneumonia. Influenza and Other Respiratory Viruses. 2011;5(6):e479-86.

53. Vilain P, Pagès F, Combes X, Marianne Dit Cassou P-J, Mougin-Damour K, Jacques-Antoine Y, et al. Health impact assessment of cyclone bejisa in reunion island (france) using syndromic surveillance. Prehospital and Disaster Medicine. 2015;30(2):137-44.

54. Noel G, Viudes G, Laporte R, Minodier P. Evaluation of the impact of pneumococcal conjugate vaccine on pediatric community-acquired pneumonia using an emergency database system. Journal of the Pediatric Infectious Diseases Society. 2017;6(2):129-33.

55. Josseran L, Nicolau J, Caillere N, Astagneau P, Brucker G. Syndromic surveillance based on emergency department activity and crude mortality: two examples. Euro surveillance. 2006;11(12):225-9.

56. Vilain P, Larrieu S, Mougin-Damour K, Marianne Dit Cassou P-J, Weber M, Combes X, et al. Emergency department syndromic surveillance to investigate the health impact and factors associated with alcohol intoxication in Reunion Island. Emergency Medicine Journal. 2017;34(6):386-90.

57. Josseran L, Fouillet A, Caillère N, Brun-Ney D, Ilef D, Brucker G, et al. Assessment of a syndromic surveillance system based on morbidity data: results from the Oscour network during a heat wave. PLoS ONE. 2010;5(8):e11984.

58. Vandentorren S, Paty AC, Baffert E, Chansard P, Caserio-Schönemann C. Syndromic surveillance during the Paris terrorist attacks. Lancet. 2016;387(10021):846-7.

59. Josseran L, Caillère N, Brun-Ney D, Rottner J, Filleul L, Brucker G, et al. Syndromic surveillance and heat wave morbidity: A pilot study based on emergency departments in France. BMC Medical Informatics and Decision Making. 2009;9(1).

60. Josseran L, Caillère N, Brun-Ney D, Filleul L, Beaujouan L, Ilèf D, et al. Performances of a syndromic surveillance system during a heat wave. Advances in Disease Surveillance 2007;4:253.

61. Josseran L, Caillère N, Brun-Ney D, Rottner J, Ilèf D, Astagneau P. Syndromic Surveillance and Heat Wave: Is it Working? Advances in Disease Surveillance 2007;4:169.

62. Josseran L, Fouillet A, Caillère N, Pascal M, Ilèf D, Astagneau P. Syndromic surveillance and climate change, a possible use? Advances in Disease Surveillance 2008;5:106.

63. Fouillet A, Fournet N, Caillère N, Musset A, Mercier L, Durand C, et al. SurSaUD® Software: A Tool to Support the Data Management, the Analysis and the Dissemination of Results from the French Syndromic Surveillance System. Online Journal of Public Health Informatics 2013; Vol 5, No 12013;5(1):e118.

64. Vilain P, Maillard O, Raslan-Loubatie J, Ahmed Abdou M, Lernout T, Filleul L. Usefulness of Syndromic Surveillance for Early Outbreak Detection in Small Islands: The Case of Mayotte. Online Journal of Public Health Informatics 2013;5(1):e149.

65. Pioche C, Larsen C, Caserio-Schonemann C, Héraud-Bousquet V. Syndromic Surveillance of Acute Liver Failure in Emergency Departments (France, 2010-2012). Online Journal of Public Health Informatics 2014;6(1):e166.

66. Vilain P, Larrieu S, Combes X, Bourdé A, Marianne dit Cassou P-J, Mougin Damour K, et al. Using a Syndromic Approach to Study Health Impact and Risk Factors of Alcohol Intoxication in Reunion Island. Online Journal of Public Health Informatics 2014;6(1):e171.

67. Fouillet A, Bousquet V, Pontais I, Gallay A, Caserio- Schönemann Cl. The French Emergency Department OSCOUR Network: Evaluation After a 10-year Existence. Online Journal of Public Health Informatics 2015; Vol 7, No 12015;7(1):e74.

68. Vilain P, Pagès Fdr, Mougin-Damour K, Combes X, Marianne Dit Cassou P-J, Antoine YJ, et al. Using an Emergency Department Syndromic Surveillance System to Assess the Impact of Cyclone Bejisa, Reunion Island. Online Journal of Public Health Informatics 2015;7(1):e171.

69. Josseran L, Gailhard I, Eboumbou B, Brucker G. Syndromic Surveillance, The First French Experience. Advances in Disease Surveillance 2006;1:37.

70. Burdet S, Fouillet A, Caillere Ng, Brun-Ney D, Desenclos J-C, Josseran Lc. Toward a predictive model of the daily ED visits in Paris area from 2007 to 2010. Emerging Health Threats Journal2011;4:60.

71. Vilain P, Bourde´ A, Cassou P-JMD, Morbidelli P, Jacques-Antoine Y, Ristor B, et al. Integrated approach of nonspecific surveillance in Re´ union Island. Emerging Health Threats Journal2011;4:155.

72. Martin A, Vilain P, Bourdé A, Combes X, Marianne dit Cassou P-J, Jacques-Antoine Y, et al. Usefulness of Syndromic Surveillance during Ultra-endurance Running Races: Example with the “Grand Raid de la Réunion” Ultra Trail. Online Journal of Public Health Informatics 2014;6(1):e79.

73. Caserio-Schönemann Cl, Sanna A, Bousquet V, Medina S, Pascal M, Delmas M-C, et al. Determinants of Daily Attendances in Emergency Departments for Asthma in the Paris Area. Online Journal of Public Health Informatics 2015;7(1):e114.

74. Pontais I, Bousquet V, Ruello M, Caserio-Schönemann Cl, Fouillet A. Factors Influencing the Stability and Quality of the French ED Surveillance System. Online Journal of Public Health Informatics 2016;8(1):e153.

75. Fouillet A, Rosine J, Bousquet V, Cassadou S, Carvalho L, Ledrans M, et al. Chikungunya Epidemic in the French Overseas Territories using Syndromic Surveillance. Online Journal of Public Health Informatics 2015;7(1):e73.

76. Vilain P, Pages F, Henrion G, Combes X, Weber M, Marianne Dit Cassou P-J, et al. Enhanced syndromic surveillance during the 9th Indian Ocean Island Games, 2015. Online Journal of Public Health Informatics 2017;9(1):e167.

77. Fougère E, Caserio-Schönemann C, Daoudi J, Fouillet A, Ruello M, Pontias I, et al. Syndromic surveillance and UEFA Euro 2016 in France – Health impact assessment. Online Journal of Public Health Informatics 2017;9(1):e171.

78. Ruello M, Pelat C, Caserio-Schönemann C, Fouillet A, Bonmarin I, Levy-Brühl D, et al. A regional approach for the influenza surveillance in France. Online Journal of Public Health Informatics 2017;9(1):e89.

79. Vilain P, Larrieu S, Cossin S, Caserio-Schönemann C, Filleul L. Wikipedia: a tool to monitor seasonal diseases trends? Online Journal of Public Health Informatics 2017;9(1):e52.

80. Fouillet A, Ruello M, Leon L, Sommen C, Marie L, Caserio-Schönemann C, et al. User-friendly Rshiny web applications for supporting syndromic surveillance analysis. Online Journal of Public Health Informatics 2017;9(1):e50.

81. Bousquet V, Vernier L, Le Strat Y, Bonmarin I, Leroy C, Raphaël M, et al. Role of Influenza in ED Visits and Hospitalizations of Adults Over 65 Years in France. Online Journal of Public Health Informatics 2016;8(1):e12.

82. Vilain P, Cossin Sb, Filleul L. Interest of Prospective Spatio-Temporal Analysis from ED Data to Detect Unusual Health Events. Online Journal of Public Health Informatics 2016;8(1):e171.

83. Vilain P, Bourde A, Marianne dit Cassou P-J, Jacques-Antoine Y, Morbidelli P, Filleul L. Syndromic Surveillance Based on Emergency Visits: A Reactive Tool for Unusual Events Detection. Online Journal of Public Health Informatics 2013;5(1):e150.

84. Hughes H, Dobney A, Fouillet A, Caserio-Schönemann C, Hughes T, Smith GE, et al. Syndromic surveillance of air pollution incidents across international borders. Online Journal of Public Health Informatics 2017;9(1):e139.

85. Dafni UG, Tsiodras S, Panagiotakos D, Gkolfinopoulou K, Kouvatseas G, Tsourti Z, et al. Algorithm for statistical detection of peaks--syndromic surveillance system for the Athens 2004 Olympic Games. MMWR Morbidity and Mortality Weekly Report 2004;53:86-94.

86. Dafni U, Golfinopoulou K, Tsiodras S, Saroglou G. Planning syndromic surveillance for the Athens 2004 Olympic games: A pilot study. Journal of Urban Health 2003;80(1):i125-i.

87. Ansaldi F, Orsi A, Altomonte F, Bertone G, Parodi V, Carloni R, et al. Syndrome surveillance and molecular epidemiology for early detection and tracing of an outbreak of measles in Liguria, Italy. Journal of Medical Virology. 2009;81(10):1807-13.

88. Ansaldi F, Orsi A, Trucchi C, De Florentiis D, Ceravolo A, Coppelli M, et al. Potential effect of PCV13 introduction on Emergency Department accesses for lower respiratory tract infections in elderly and at risk adults. Human vaccines & immunotherapeutics. 2015;11(1):166-71.

89. Ansaldi F, Orsi A, Altomonte F, Bertone G, Parodi V, Carloni R, et al. Emergency department syndromic surveillance system for early detection of 5 syndromes: a pilot project in a reference teaching hospital in Genoa, Italy. Journal of preventive medicine and hygiene. 2008;49(4):131-5.

90. De Florentiis D, Parodi V, Orsi A, Rossi A, Altomonte F, Canepa P, et al. Impact of influenza during the post-pandemic season: epidemiological picture from syndromic and virological surveillance. Journal of preventive medicine and hygiene. 2011;52(3):134-6.

91. Guasticchi G, Giorgi Rossi P, Lori G, Genio S, Biagetti F, Gabriele S, et al. Syndromic surveillance: Sensitivity and positive predictive value of the case definitions. Epidemiology and Infection. 2009;137(5):662-71.

92. Aung M. Syndromic Surveillance In Major Sporting Event - Jamaican Experience. Advances in Disease Surveillance 2007;4; :144.

93. Ahn S, Lee JH, Kim W, Lim KS. Analysis of the korean emergency department syndromic surveillance system: mass type acute diarrheal syndrome. Healthcare informatics research. 2010;16(3):177-84.

94. Kam HJ, Choi S, Cho JP, Min YG, Park RW. Acute diarrheal syndromic surveillance: effects of weather and holidays. Applied clinical informatics. 2010;1(2):79-95.

95. Cho JP, Kim JS, Yoo IS, Ahn MY, Wang SJ, Hur T, et al. Syndromic surveillance based on the emergency department in Korea. Journal of Urban Health2003;80(1):i124-i5.

96. McLeod M, White P, Read D, Mason K. The 2005 Wellington influenza outbreak: Syndromic surveillance of Wellington Hospital Emergency Department activity may have provided early warning. Australian and New Zealand Journal of Public Health. 2009;33(3):289-94.

97. Hishamuddin P. Evaluation of Syndromic Surveillance Systems in Singapore. Online Journal of Public Health Informatics 2014;6(1):e142.

98. Schrell S, Ziemann A, Garcia-Castrillo Riesgo L, Rosenkötter N, Llorca J, Popa D, et al. Local implementation of a syndromic influenza surveillance system using emergency department data in Santander, Spain. Journal of public health (Oxford, England). 2013;35(3):397-403.

99. Ziemann A, Rosenkötter N, Garcia-Castrillo Riesgo L, Schrell S, Kauhl B, Vergeiner G, et al. A concept for routine emergency-care data-based syndromic surveillance in Europe. Epidemiology and Infection. 2014;142(11):2433-46.

100. Wu T-SJ, Shih F-YF, Yen M-Y, Wu J-SJ, Lu S-W, Chang KC-M, et al. Establishing a nationwide emergency department-based syndromic surveillance system for better public health responses in Taiwan. BMC public health. 2008;8:18.

101. Lo Y-C, Chuang J-H, Kuo H-W, Huang W-T, Hsu Y-F, Liu M-T, et al. Surveillance and vaccine effectiveness of an influenza epidemic predominated by vaccine-mismatched influenza B/Yamagata-lineage viruses in Taiwan, 2011-12 season. PLoS ONE. 2013;8(3):e58222.

102. Chang C, Chan T-C, Lai Y-M, Fang C-C, Shih F-Y, Yen M-Y, et al. Establishment of public-access syndromic surveillance system in Taipei City, Taiwan. Emerging Health Threats Journal2011;4:24.

103. King C-C, Shih F-Y, Yen M-Y, Hu F-C, Wu J-S, Chang F-K, et al. Syndromic Surveillance of Infectious Diseases in Taiwan — Before and After the Challenges of Severe Acute Respiratory Syndrome (SARS). MMWR: Morbidity & Mortality Weekly Report 2004;53:245.

104. Hsiao C-Y, Wu T-SJ, Shih F-YF, Yen M-Y, Tipayamongkholgul M, Pan M-L, et al. Establishing a Syndromic Surveillance System Using Chief-Complaint Data at Emergency Department of One Regional Hospital in Taipei City to Detect Infectious Disease Outbreaks. Advances in Disease Surveillance 2007;2:107.

105. King C-C, Wu T-S, Hsiao C-Y, Shih F-YF, Yen M-Y, Liu D-M, et al. Comparison of Chief-Complaint vs ICD-9 Data Used in an Emergency Departmentbased Hospital Syndromic Surveillance System in Metropolitan Taipei, Taiwan. Advances in Disease Surveillance 2007;2:202.

106. King C-C, Wu T-SJ, Yen M-Y, Huang C, Hsiao J-Y, Kao C-L, et al. INTEGRATED SYNDROMIC AND VIROLOGIC SURVEILLANCE SYSTEMS FROM TAIWAN’S EXPERIENCES IN FACING GLOBAL CHALLENGES OF EMERGING INFECTIOUS DISEASES Advances in Disease Surveillance 2008;5:109.

107. Elliot AJ, Smith S, Dobney A, Thornes J, Smith GE, Vardoulakis S. Monitoring the effect of air pollution episodes on health care consultations and ambulance call-outs in England during March/April 2014: a retrospective observational analysis. Environmental Pollution. 2016;214:903-11.

108. Hughes HE, Morbey R, Hughes TC, Locker TE, Pebody R, Green HK, et al. Emergency department syndromic surveillance providing early warning of seasonal respiratory activity in England. Epidemiology and Infection. 2016;144(5):1052-64.

109. Smith S, Elliot AJ, Hajat S, Bone A, Smith GE, Kovats S. Estimating the burden of heat illness in England during the 2013 summer heatwave using syndromic surveillance. Journal of Epidemiology & Community Health. 2016;70(5):459-65.

110. Todkill D, Hughes HE, Elliot AJ, Morbey RA, Edeghere O, Harcourt S, et al. An observational study using English syndromic surveillance data collected during the 2012 London Olympics - what did syndromic surveillance show and what can we learn for future mass-gathering events? Prehospital and Disaster Medicine. 2016;31(6):628-34.

111. Smith S, Elliot AJ, Hajat S, Bone A, Bates C, Smith GE, et al. The impact of heatwaves on community morbidity and healthcare usage: a retrospective observational study using real-time syndromic surveillance. International Journal of Environmental Research and Public Health. 2016;13(1):132-.

112. Bawa Z, Elliot AJ, Morbey RA, Ladhani S, Cunliffe NA, O'Brien SJ, et al. Assessing the likely impact of a rotavirus vaccination program in England: the contribution of syndromic surveillance. Clinical Infectious Diseases. 2015;61(1):77-85.

113. Morbey RA, Elliot AJ, Charlett A, Ibbotson S, Verlander NQ, Leach S, et al. Using public health scenarios to predict the utility of a national syndromic surveillance programme during the 2012 London Olympic and Paralympic Games. Epidemiology and Infection. 2014;142(5):984-93.

114. Hughes HE, Morbey R, Hughes TC, Locker TE, Shannon T, Carmichael C, et al. Using an Emergency Department Syndromic Surveillance System to investigate the impact of extreme cold weather events. Public Health. 2014;128(7):628-35.

115. Hughes HE, Hughes TC, Haile A, Smith GE, McCloskey B, Elliot AJ. Syndromic Surveillance Revolution? Public Health Benefits of Modernizing the Emergency Care Patient Health Record in England. Public Health Reports. 2017;132(1).

116. Elliot AJ, Hughes HE, Hughes TC, Locker TE, Brown R, Sarran C, et al. The impact of thunderstorm asthma on emergency department attendances across London during July 2013. Emergency Medicine Journal. 2014;31(8):675-8.

117. Elliot AJ, Morbey R, Edeghere O, Lake IR, Colón-González FJ, Vivancos R, et al. Developing a Multidisciplinary Syndromic Surveillance Academic Research Program in the United Kingdom: Benefits for Public Health Surveillance. Public Health Reports. 2017;132(1).

118. Elliot AJ, Hughes HE, Hughes TC, Locker TE, Shannon T, Heyworth J, et al. Establishing an emergency department syndromic surveillance system to support the London 2012 Olympic and Paralympic Games. Emergency medicine journal : EMJ. 2012;29(12):954-60.

119. Newitt S, Elliot AJ, Morbey R, Durnall H, Pietzsch ME, Medlock JM, et al. The use of syndromic surveillance to monitor the incidence of arthropod bites requiring healthcare in England, 2000-2013: a retrospective ecological study. Epidemiology and infection. 2016;144(11):2251-9.

120. Thomas SL, Walker JL, Fenty J, Atkins KE, Elliot AJ, Hughes HE, et al. Impact of the national rotavirus vaccination programme on acute gastroenteritis in England and associated costs averted. Vaccine. 2017;35(4):680-6.

121. Severi E, Heinsbroek E, Watson C, Catchpole M. Infectious disease surveillance for the London 2012 Olympic and Paralympic Games. Eurosurveillance. 2012;17(31).

122. Morbey R, Elliot AJ, Zambon M, Pebody R, Smith GE. Interpreting specific and general respiratory indicators in syndromic surveillance. Online Journal of Public Health Informatics 2017;9(1):e19.

123. Hughes H, Morbey R, Hughes T, Locker T, Smith G, Elliot A. Syndromic Surveillance of Respiratory Pathogens using Routine ED Data in England. Online Journal of Public Health Informatics 2015;7(1):e81.

124. Kass-Hout TA, Xu Z, McMurray P, Park S, Buckeridge DL, Brownstein JS, et al. Application of change point analysis to daily influenza-like illness emergency department visits. Journal of the American Medical Informatics Association. 2012;19(6):1075-81.

125. Olson DR, Paladini M, Buehler JW, Mostashari F, group tDW. Review of the ISDS Distributed Surveillance Taskforce for Real-time Influenza Burden Tracking & Evaluation (DiSTRIBuTE) Project 2007/08 Influenza Season Proof-of-concept Phase Advances in Disease Surveillance 2008;5:185.

126. Paladinia M, Pendarvisb J, Murraya EL, Gunnb J, Olson D. A Comparison of Locally Developed Influenza-like Syndrome Definitions Using Electronic Emergency Department Data in Boston and New York City Advances in Disease Surveillance 2008;5:50.

127. Painter I, Eaton J, Lober B. Using Change Point Detection for Monitoring the Quality of Aggregate Data. Online Journal of Public Health Informatics 2013;5(1):e186.

128. Olson D, Baer A, Paladini M, Burkom H, Group IDW. Adopting a common influenza-like illness syndrome across multiple health jurisdictions. Emerging Health Threats Journal2011;4:110.

129. Olson D, Painter I, Group IDW. Monitoring winter-seasonal acute gastroenteritis emergency department visits by age. Emerging Health Threats Journal2011;4:111.

130. Painter I, Eaton J, Olson D, Revere D, Lober W. How good is your data? Emerging Health Threats Journal2011;4:114.

131. Painter I, Eaton J, Olson D, Lober W, Revere D. Visualizing data quality: tools and views. Emerging Health Threats Journal2011;4:115.

132. Olson D, Baer A, Paladini M, Burkom H, Revere D, Painter I, et al. Evaluating a standard influenza-like illness syndrome definition across multiple sites in the distribute project: The ‘ILI-s’ Pilot. Emerging Health Threats Journal2011;4(1):s48.

133. Kniss K, Benoit S, McIntyre A, Buckeridge D, Brownstein J, Brammer L, et al. Comparison of Distribute and ILINet for national influenza surveillance. Emerging Health Threats Journal2011;4(1):s135.

134. White JR, Berisha V, Lane K, Ménager H, Gettel A, Braun CR, et al. Evaluation of a Novel Syndromic Surveillance Query for Heat-Related Illness Using Hospital Data From Maricopa County, Arizona, 2015. Public health reports. 2017;132(1).

135. Coates RJ, Pérez A, Baer A, Zhou H, English R, Coletta M, et al. National and Regional Representativeness of Hospital Emergency Department Visit Data in the National Syndromic Surveillance Program, United States, 2014. Disaster medicine and public health preparedness. 2016;10(4):562-9.

136. Wiedeman C, Shaffner J, Squires K, Leegon J, Murphree R, Petersen PE. Monitoring Out-of-State Patients During a Hurricane Response Using Syndromic Surveillance -- Tennessee, 2017. MMWR: Morbidity & Mortality Weekly Report 2017;66(49):1364-5.

137. Gould DW, Walker D, Yoon PW. The Evolution of BioSense: Lessons Learned and Future Directions. Public Health Reports. 2017;132(1).

138. DeYoung K, Chen Y, Beum R, Askenazi M, Zimmerman C, Davidson AJ. Validation of a Syndromic Case Definition for Detecting Emergency Department Visits Potentially Related to Marijuana. Public health reports. 2017;132(4):471-9.

139. Ayala A, Berisha V, Goodin K, Pogreba-Brown K, Levy C, McKinney B, et al. Public health surveillance strategies for mass gatherings: Super Bowl XLIX and related events, Maricopa County, Arizona, 2015. Health Security. 2016;14(3):173-84.

140. Xing J, Burkom H, Tokars J. Method selection and adaptation for distributed monitoring of infectious diseases for syndromic surveillance. Journal of Biomedical Informatics. 2011;44(6):1093-101.

141. Kass-Hout TA, Buckeridge D, Brownstein J, Xu Z, McMurray P, Ishikawa CKT, et al. Self-reported fever and measured temperature in emergency department records used for syndromic surveillance. Journal of the American Medical Informatics Association. 2012;19(5):775-6.

142. Rezny S, Hoferka S. An R Script for Assessment of Data Quality in the BioSense Locker Database. Online Journal of Public Health Informatics 2016;8(1):e156.

143. Thoburn KK, Miller JR, Tokars JI, Bradley C, Zomer D. The New York State BioSense Sentinel Alert Experience. Advances in Disease Surveillance 2006;1:68.

144. Dey AN, Ma H, Asatryan A, English R, Tokars J. Identifying Fractures in BioSense Radiology Reports Advances in Disease Surveillance 2007;4:159.

145. Lipskiy N, Tokars JI, Copeland J, English R, Patel N. Visits with Nontyphoidal Salmonella Infections Reported to the BioSense System, 2006-2007. Advances in Disease Surveillance 2007;4:173.

146. Martin C, Tokars J, Fowlkes A, Panozzo C. Use of Final Diagnosis Data for Surveillance of Respiratory Syncytial Virus Advances in Disease Surveillance 2007;4:176.

147. Martin CA, Tokars J, Ma H. ICD-9 CM Based Sub-Syndrome Distributions in BioSense Hospital Data. Advances in Disease Surveillance 2007;2:110.

148. Podgornik MN, English R, Tokars JI. Correlation between Real-Time BioSense Influenza Indicators and Data from the U.S. Influenza Sentinel Physicians Surveillance Network Advances in Disease Surveillance 2007;4:109.

149. Rainisch G, Martin C, Tokars J. The Use of BioSense Data for Surveillance of Gastrointestinal Illness. Advances in Disease Surveillance 2007;4:185.

150. Dey AN, Hicks P, Tokars JI. Identifying Clusters of Falls During the 2007-08 Winter Season in the BioSense System. Advances in Disease Surveillance 2008;5:101.

151. Hales C, English R, McMurray P, Tokars J. The BioSense Influenza Module Advances in Disease Surveillance 2008;5:36.

152. Lei WE, Benoit S, Rainisch G, Tokars J. Burns Reported to the BioSense System During the Independence Day Holiday. Advances in Disease Surveillance 2008;5:111.

153. Minor P, English R, Tokars J. Representativeness of Emergency Department Data Reported to the BioSense System. Advances in Disease Surveillance 2008;5:182.

154. Mohammed S, Phillips K, Rainisch G, Minor P, Tokars J. Automated Monitoring of Asthma Using the BioSense System. Advances in Disease Surveillance 2008;5:119.

155. Phillips K, Rainisch G, Tokars J. Automated Monitoring of Exposures Using the BioSense System Advances in Disease Surveillance 2008;5:188.

156. Xing J, Burkom H, Tokars J. Comparison of Regression Models with Modified Time Series Methods for BioSurveillance. Advances in Disease Surveillance 2008;5:72.

157. Arboleda N, Tokars J, English R, McMurray P. BioSense Real-Time Data Initiative: Improving Emergency Preparedness - Monitoring Injury sub-syndromes. Advances in Disease Surveillance 2007;4:142.

158. Martin C, Tokars J, Rainisch G, Burkholder J, Crosby K. Criteria for Prioritizing Statisitical Anomalies Identified in BioSense. Advances in Disease Surveillance 2007;4:174.

159. Hicks P, Edwards A, English R, Tokars JI. BioSense Participation by Non-Federal Hospitals. Advances in Disease Surveillance 2008;5:38.

160. Dey A, Park S, Miller M, Akaka L, McMurray P, Hicks P, et al. Postdisaster surveillance following the Tsunami in Japan: BioSense 2011. Emerging Health Threats Journal2011;4:41.

161. McIntyre A, Finelli L, Burkom H, Benoit S, Kniss K, Kass-Hout T. Quantifying the relationship between influenza-related emergency department visits and hospital admissions in BioSense. Emerging Health Threats Journal2011;4(1):s99.

162. Dey AN, Grigoryan A, Park S, Benoit S, Kass-Hout T. Mental Illness and Co-morbid Conditions: BioSense 2008 - 2011. Online Journal of Public Health Informatics 2013;5(1):e59.

163. Burrer S, Burkom H, Okunseri C, Barker L, Robison V. Nontraumatic Oral Health Classification for Alternative Use of Syndromic Data. Online Journal of Public Health Informatics 2013;5(1):e58.

164. Baber J, Feist M. Utility of Outpatient Syndromic Data for Monitoring Influenza-like Illness. Online Journal of Public Health Informatics 2017;9(1):e51.

165. Baber J, Miller T. Using Sydromic Surveillance to Track E-cigarette Related Emergency Department Visits. Online Journal of Public Health Informatics 2017;9(1):e144.

166. Burke A, Jackson D, Nakashima AK. An Evaluation of the Biosense 2.0 “Poisoning by Medicines” Syndrome Using Chief-Complaint Data in Utah. Online Journal of Public Health Informatics 2015;7(1):e10.

167. Burke A, Jackson D, Nakashima AK. An Ecological Analysis of the Impact of Temperature Inversion on Emergency Department Visits for Respiratory Syndromes and Subsyndromes using BioSense 2.0 Frontend Data. Online Journal of Public Health Informatics 2015;7(1):e9.

168. Yun LW, Beum R, Askenazi M, Chen Y, McEwen D, Maravi M, et al. Using Emergency Department Data for Detection of a Synthetic Marijuana Outbreak. Online Journal of Public Health Informatics 2015;7(1):e178.

169. Yoon P, Coletta M. Update on the CDC National Syndromic Surveillance Program. Online Journal of Public Health Informatics 2016;8(1):e183.

170. Stein ZM. Creation of a Kansas Spring Extreme Weather Syndrome Definition and Unique Records. Online Journal of Public Health Informatics 2017;9(1):e128.

171. Bova M, Ergas R. Early effect of validation efforts of Massachusetts syndromic surveillance data. Online Journal of Public Health Informatics 2017;9(1):e35.

172. DeYoung KH, Beum R, Chen Y, Maravi M, Yun LW, Askenazi M, et al. Evaluation of Case Detection of Marijuana-Related Emergency Department Visits. Online Journal of Public Health Informatics 2016;8(1):e17.

173. Anderson TC, Yusuf H, McCarthy A, Trivers K, Hicks P, Coletta M, et al. Administrative and syndromic surveillance data can enhance public health surveillance. Online Journal of Public Health Informatics 2017;9(1):e165.

174. Betancourt JA, Hakre S, Polyak CS, Pavlin JA. Evaluation of ICD-9 codes for syndromic surveillance in the Electronic Surveillance System for the Early Notification of Community-based Epidemics. Military Medicine. 2007;172(4):346-52.

175. Pattie DC, Atherton MJ, Savory DJ, Savage GE, Cox KL. ESSENCE Version 2.0: The Department of Defense’s Worldwide Syndromic Surveillance System Receives Several Enhancements. Advances in Disease Surveillance 2008;5:51.

176. Lucero C, Schirmer P, Oda G, Holodniy M. Surveillance for nationally notifiable infectious conditions using ICD-9-CM diagnosis codes in the VA ESSENCE biosurveillance system. Emerging Health Threats Journal 2011;4(1):s97.

177. Rangel M, Martinello R, Lucero C, Oda G, Holodniy M, Peterson M, et al. Adapting a syndromic biosurveillance system to monitor veterans’ health impact associated with the gulf coast oil spill. Emerging Health Threats Journal 2011;4(1):s138.

178. Oda G, Ryono R, Lucero-Obusan CA, Schirmer P, Holodniy M. Carbon Monoxide Poisoning in the Veterans Health Administration, 2010 - 2016. Online Journal of Public Health Informatics 2017; Vol 9, No 12017;9(1):e126.

179. Fleischauer AT, Silk BJ, Schumacher M, Komatsu K, Santana S, Vaz V, et al. The validity of chief complaint and discharge diagnosis in emergency department-based syndromic surveillance. Academic Emergency Medicine. 2004;11(12):1262-7.

180. Fleischauer AT, Silk B, Schumacher M, Komatsu K, Santana S, Vaz V, et al. Evaluation and Validity of Chief Complaints and Discharge Diagnoses in a Drop-In Syndromic Surveillance System. MMWR: Morbidity & Mortality Weekly Report 2004;53:238.

181. Arboleda N, Fleischauer AT, Sejvar J, Diggs A, Schumacher M, Santana S, et al. An Emergency Department Based Syndromic Surveillance System for Meningitis and Encephalitis, Maricopa County, AZ 2004. Advances in Disease Surveillance 2006;1:4.

182. Schumacher M, Nohre L, Santana S. Partial evaluation of a drop-in bioterrorism surveillance system in phoenix, Arizona. Journal of Urban Health 2003;80(1):i118-i.

183. Kajita E, Luarca MZ, Wu H, Hwang B, Mascola L. Harnessing Syndromic Surveillance Emergency Department Data to Monitor Health Impacts During the 2015 Special Olympics World Games. Public Health Reports. 2017;132(1):99s-105s.

184. Belden J, Araki P, Croker C, Sharip A, Velikina R, Hwang B, et al. Syndromic Surveillance Signal Investigation in Los Angeles County. Advances in Disease Surveillance 2006;1:5.

185. Croker C, Sharip A, Villacorte F, Tormey M, Kim M, Hwang B, et al. Evaluating the Effectiveness of Using Syndromic Surveillance to Identify a Neuro-Invasive Disease Outbreak in Los Angeles County Advances in Disease Surveillance 2006;1:18.

186. Kajita E, Sharip A, Araki P, Hwang B. Emergency Department Syndromic Surveillance and Population-Based Health Monitoring in Los Angeles County. Advances in Disease Surveillance 2007;4:170.

187. Sharip A, Hwang B, Wu H, Yee D, Toyota C, Croker C, et al. Automated Syndromic Surveillance System in Los Angeles County Advances in Disease Surveillance 2007;2:27.

188. Sharip A, Kajita E, Jones M, Hwang B. Health Effects of LA County Wildfires in October 2007 Advances in Disease Surveillance 2008;5:129.

189. Kajita E, Araki P, Luarca M, Hwang B. Monitoring the Impact of Heat Waves with Emergency Service Utilization Data in Los Angeles County. Online Journal of Public Health Informatics 2013;5(1):3153.

190. Oyong K, Kajita E, Araki P, Luarca M, Hwang B. Validation of Los Angeles County Department of Public Health Respiratory Syndrome using Electronic Health Records. Online Journal of Public Health Informatics 2014;6(1):e81.

191. Araki P, Kajita E, Oyong K, Luarca MZ, Hwang B, Mascola L. A Comparison of Fever Classified Chief Complaints and Diagnoses with Recorded Body Temperatures. Online Journal of Public Health Informatics 2015;7(1):e105.

192. Kajita E, Luarca MZ, Chiang C, Wu H, Hwang B. Syndromic Surveillance of Emergency Department Visits for the 2015 Special Olympics. Online Journal of Public Health Informatics 2016;8(1):e129.

193. Sharip A, Monroe J, Croker C, Kim M, Hwang B, Aller R, et al. Preliminary Analysis of SaTScan's Effectiveness to Detect Known Disease Outbreaks Using Emergency Department Syndromic Data in Los Angeles County. Advances in Disease Surveillance 2006;1:83.

194. Luarca M, Kajita E, Faustino C, Jones M, Hwang B. Using syndromic surveillance to assist in a meningitis outbreak investigation. Emerging Health Threats Journal 2011;4:78.

195. Viola R, Luarca MZ, Kajita E, Lim M, Hwang B. Monitoring the 2016 LA County Sand Fire with Multiple Early Detection Systems. Online Journal of Public Health Informatics 2017;9(1):e135.

196. Bravata DM, Rahman MM, Luong N, Divan HA, Cody SH. A comparison of syndromic incidence data collected by triage nurses in Sata Clara county with regional infectious disease data. Journal of Urban Health 2003;80(1):i122-i.

197. Thelen B, French NH, Koziol BW, Billmire M, Owen RC, Johnson J, et al. Modeling acute respiratory illness during the 2007 San Diego wildland fires using a coupled emissions-transport system and generalized additive modeling. Environmental Health: A Global Access Science Source. 2013;12(1).

198. Johnson JM, Hicks L, McClean C, Ginsberg M. Leveraging Syndromic Surveillance During the San Diego Wildfires, 2003. MMWR: Morbidity & Mortality Weekly Report 2005;54:190.

199. McClean C, Johnson J, Colanter B, Ginsberg M. Too Many Signals? Frequency and Quantitative Descriptions of Detected Events. Advances in Disease Surveillance 2006;1:49.

200. Colanter BH, Johnson JM, Maroufi A, Ginsberg MM, Nelson JA. Using Syndromic Surveillance Data for Enhanced Case-Capture of Conditions of Public Health Interest. Advances in Disease Surveillance 2007;4:155.

201. Johnson J, Ginsberg M, French N, Thelen B, Koziol B. Use of Syndromic Surveillance Information for Expanded Assessment of Wildfire Disaster. Online Journal of Public Health Informatics 2013;5(1):e95.

202. Johnson J, McClean C, Poggemeyer K, Ginsberg M. Application of bioterrorism surveillance methods in San Diego County. Journal of Urban Health 2003;80(1):i137.

203. Chen Y, Askenazi M, DeYoung KH, Albanese B, Yun LW, Hockenberry T. ED Visits Related to Marijuana Exposures in the Denver Metropolitan Area of Colorado. Online Journal of Public Health Informatics 2016;8(1):e99.

204. Foldy SL, Barthell E, Silva J, Biedrzycki P, Howe D, Erme M, et al. SARS Surveillance Project -- Internet-Enabled Multiregion Surveillance for Rapidly Emerging Disease. MMWR: Morbidity & Mortality Weekly Report 2004;53:215-20.

205. Dembek ZF, Carley K, Hadler J. Guidelines for Constructing a Statewide Hospital Syndromic Surveillance Network. MMWR: Morbidity & Mortality Weekly Report 2005;54:21-4.

206. Purviance KM, Siniscalchi AJ, Hadler JL. Surveillance for Influenza Using the Emergency Department Syndromic Surveillance and Hospital Admissions Syndromic Surveillance Systems, Connecticut, 2004-2007. Advances in Disease Surveillance 2007;4:184.

207. Purviance KM, Siniscalchi AJ, Hadler JL. Use of the Connecticut Emergency Department Syndromic Surveillance System for Situational Awareness During Public Health Events. Advances in Disease Surveillance 2008;5:125.

208. Soto K, Krasnitski J, Rabatsky-Ehr T, Cartter M. When it Rains it Pours: Real-time Situational Awareness for Two Weather Emergencies in Connecticut. Online Journal of Public Health Informatics 2013;5(1):e97.

209. Soto K, Krasnitski J, Phan Q, Rabatsky-Ehr T, Cartter M. Evaluation of the Connecticut hospital emergency department syndromic surveillance system for monitoring of community gastrointestinal illness. Emerging Health Threats Journal 2011;4:142.

210. Desy K, Purviance K, Cartter M, Hadler J. Relationship between neighborhood poverty and emergency department utilization for fever/flu syndrome. Emerging Health Threats Journal 2011;4(1):s75.

211. Purviance K, Brockmeyer J, Krasnitski J, Siniscalchi A, Cartter M, Hadler J. Experience Using Syndromic Surveillance Systems During the Novel H1N1 Influenza Outbreak, Connecticut, 2009. ISDS; Miami, Fl 2009.

212. Siniscalchi AJ, Andrews MT, Purviance KM, Esponda BL, Brennan T, Nelson RS, et al. Yes We Can! Use of Multiple Syndromic Surveillance Systems for Detection of Post Inauguration and Late Season Influenza Events. ISDS; Miami, Fl 2009.

213. Klekamp BG, Bodager D, Matthews SD. Use of Surveillance Systems in Detection of a Ciguatera Fish Poisoning Outbreak - Orange County, Florida, 2014. MMWR: Morbidity & Mortality Weekly Report. 2015;64(40):1142-4.

214. Hopkins RS, Kite-Powell A, Goodin K, Hamilton JJ. The Ratio of Emergency Department Visits for ILI to Seroprevalence of 2009 Pandemic Influenza A (H1N1) Virus Infection, Florida, 2009. PLoS Currents. 2014;6.

215. Kite-Powell A, Ball LB, Hopkins RS. Use of Syndromic Surveillance of Emergency Room Chief Complaints for Enhanced Situational Awareness during Wildfires, Florida, 2008. Advances in Disease Surveillance 2008;5:110.

216. Thomas MC, Atrubin D, Hamilton J. Assessing Best Practices for Grouping and Analyzing Urgent Care Center (UCC) and Emergency Department (ED) Data Sources within Syndromic Surveillance Systems. Online Journal of Public Health Informatics 2014;6(1):e122.

217. Wiese M, Atrubin D, McDougle W, Lewis J. Effectiveness of Using a Chief Complaint and Discharge Diagnosis Query in ESSENCE-FL to Identify Possible Tuberculosis Patients and Contacts in Hillsborough County, Florida. Online Journal of Public Health Informatics 2014;6(1):e36.

218. Rubino H, Atrubin D, Hamilton JJ. Monitoring Respiratory Syncytial Virus Regionally In Children Aged < 5 Years Old Using Emergency Department and Urgent Care Center Chief Complaint Data in Florida’s Syndromic Surveillance System, Week 1, 2010 - Week 32, 2014. Online Journal of Public Health Informatics 2015;7(1):e50.

219. Atrubin D, Bowden S, Hamilton JJ. Using Syndromic Surveillance to Rapidly Describe the Early Epidemiology of Flakka Use in Florida, June 2014 – August 2015. Online Journal of Public Health Informatics 2016;8(1):e20.

220. Culpepper AB, Atrubin D, Hamilton JJ, Cui D. Day of Week Analysis of Myocardial Infarctions Using ESSENCE-FL Emergency Department Data. Online Journal of Public Health Informatics 2016;8(1):e57.

221. Azarian T, Kite-Powell A, Zaheer S. Syndrome evaluation of individuals reporting emergency department utilization for notifiable disease and conditions. Emerging Health Threats Journal 2011;4(1):s63.

222. Kite-Powell A, Hamilton J, Wojcik R, Loschen W, Hopkins R. Florida’s ESSENCE systemFfrom syndromic surveillance to routine epidemiologic analysis across syndromic and non-syndromic data sources. Emerging Health Threats Journal 2011;4(1):s89.

223. Kite-Powell A, Hamilton J, Eisenstein L, Hopkins R. Public health surveillance after the Haiti earthquake: the Florida experience. Emerging Health Threats Journal 2011;4(1):s33.

224. Mulay P, Watkins S, Blackmore C. Utilization of Florida poison control data and Emergency Department chief complaint data to identify clusters of carbon monoxide poisoning. Emerging Health Threats Journal 2011;4(1):s41.

225. Thomas MC, Atrubin D, Hamilton JJ. Impact of Patient Self-Registration in Emergency Departments on Syndromic Surveillance Data. Online Journal of Public Health Informatics 2015;7(1):e93.

226. Munroe J, Straver R, Rubino H, Pritchard S, Atrubin D, Hamilton JJ. MERS PUI Surveillance and Restrospective Identification in ESSENCE-FL, 2013-2015. Online Journal of Public Health Informatics 2017;9(1):e113.

227. Clark CR, Wiese M. Analysis of Daily Enhanced Syndromic Surveillance in Hillsborough County, FL, 2015. Online Journal of Public Health Informatics 2017;9(1):e93.

228. Culpepper AB, Atrubin D, Hamilton JJ. The Evaluation of Triage Notes Using ESSENCE-FL for Active Case Finding of Zika. Online Journal of Public Health Informatics 2017;9(1):e23.

229. Atrubin D. Using ESSENCE-FL for Situational Awareness after National Reports of Increased Enterovirus D68 (EV-D68) Infections with Severe Outcomes, September 2014. Online Journal of Public Health Informatics 2015;7(1):e107.

230. Eisenstein L, Kite-Powell A, Hamilton J, Azarian T. Syndromic Surveillance of Pregnancy and Influenza-Like Illness from Emergency Department Chief Complaints during Pandemic H1N1 Influenza Compared to Seasonal Influenza, Florida 2008-2009. ISDS; Miami, Fl 2009.

231. Kite-Powell A, Livengood J. Use of Syndromic Surveillance during a South Florida Mass Migration Exercise, Broward County, 2007. Advances in Disease Surveillance 2007;4:171.

232. Kite-Powell A, Livengood J. Syndromic Surveillance of Emergency Department Chief Complaints postHurricane Wilma, Broward County, Florida 2005 Advances in Disease Surveillance 2007;2:157.

233. Sniegoski C, Loschen W, Dearth S, Gibson J, Lombardo J, Wade M, et al. Super Bowl Surveillance: A Practical Exercise in Inter-Jurisdictional Public Health Information Sharing. Advances in Disease Surveillance 2007;4:195.

234. Azarian T, Zaheer S, Winn S, Rios L. BioDefend™ Syndromic Surveillance System Influenza Activity Detection. Advances in Disease Surveillance 2007;4:231.

235. Azarian T, Zaheer S, Winn S, Kite-Powell A. Comparison of Influenza-like Illness Syndrome Classification Between Two Syndromic Surveillance Systems. Advances in Disease Surveillance 2008;5:1.

236. Winn SK, Isaac T, Harmon RG. A County Health Department’s Analysis and Dissemination Algorithm of a Neurological Syndrome Aberration to Respective Community Stakeholders. Advances in Disease Surveillance 2008;5:137.

237. Zaheer S, Winn S, Perry J, Minden V. Implementation of the BioDefend Syndromic Surveillance System: Electronic Format versus Web-Based Data Entry. Advances in Disease Surveillance 2007;2:30.

238. Azarian T, Kite-Powell A, Zaheer S. Improvement of epidemiology business processes through the evolution of biosurveillance. Emerging Health Threats Journal 2011;4:9.

239. Richardson D, Paladini M, Azarian T, Baer A, Rennick M, Weiss D. Increased emergency department presentations for head trauma following media coverage of a fatal epidural hematoma. Emerging Health Threats Journal 2011;4(1):s51.

240. Kintz J, Gregos E, Atrubin D, Sanchez J. Syndromic Tracking and Reporting System — Overview and Example. MMWR: Morbidity & Mortality Weekly Report 2004;53:246.

241. Zhu Y, Wang W, Atribun D, Carrubba C, Kintz J, Elliot G. A Syndrome Track and Reporting System in Hillsborough County Florida: Findings from a Systematic Evaluation. Advances in Disease Surveillance 2007;2:221.

242. Borroto-Ponce R, Zhang G, Leguen F, Sneed R, Micieli C. Catchment Area of Miami Children’s Hospital Emergency Room - Implications for Syndromic Surveillance in Miami-Dade County. Advances in Disease Surveillance 2007;2:194.

243. Micieli C, Sneed R, Erin OC, Borroto-Ponce R, Zhang G. Dual monitoring of ILI syndrome using the ESSENCE system. Advances in Disease Surveillance 2007;2:160.

244. Micieli C, Sneed R, O’Connell E, Borroto-Ponce R, Zhang G, Leguen F. Utility of the ESSENCE system for surveillance of Influenza-like Illness (ILI) in Miami-Dade County. Advances in Disease Surveillance 2007;2:112.

245. O’Connell E, Leguen F, Zhang G, Micieli C, Sneed R, Ponce-Borroto R. Comparison of ICD-9-Coded Chief Complaints and Diagnoses for Identifying Gastrointestinal Syndrome Using ESSENCE. Advances in Disease Surveillance 2007;2:210.

246. Zhang G, O’Connell E, Bustamante M, Rodriguez D, Borroto-Ponce R, Leguen F. Comparison of 2005-2007 Influenza-Like Illness Observed From Emergency Department Visits in Miami-Dade County. Advances in Disease Surveillance 2007;4:272.

247. Zhang G, O’Connell E, Leguen F, Bustamante M, Rodriguez D, Borroto-Ponce R. Use of Epidemiological Knowledge to Create Syndromic Surveillance Reports. Advances in Disease Surveillance 2007;4:211.

248. Zhang G, Sneed R, Leguen F, Cutié LA, Micieli C, Borroto-Ponce R, et al. Use of Syndromic Data for Surveillance of Hurricane-Related Injuries in Miami-Dade County, FL. Advances in Disease Surveillance 2007;2:174.

249. Borroto R, Zhang G. Socio-demographic and temporal patterns of Emergency Department patients who do not reside in Miami-Dade County, 2007. Advances in Disease Surveillance 2008;5:5.

250. O’Connell E, Zhang G, Llau A. Substance Abuse Among Youth in Miami-Dade County, 2005-2007. Advances in Disease Surveillance 2008;5:184.

251. Zhang G, Llau A, Suarez J, O'Connell E, Rico E, Borroto-Ponce R, et al. Using ESSENCE to Track a Gastrointestinal Outbreak in a Homeless Shelter in Miami-Dade County, 2008. Advances in Disease Surveillance 2008;5:139.

252. Rodriguez D, Zhang G, Leguen F, O'Connell E, Bustamante M. Using Public School Absentee Data to Enhance Snydromic Surveillance in Miami-Dade County, 2007. Advances in Disease Surveillance 2007;4:188.

253. O'Connell E, Zhang G, Llau A, Leguen F. Innovative Uses for ESSENCE to Improve Standard Communicable Disease Reporting Practices in Miami-Dade County. Advances in Disease Surveillance 2008;5:122.

254. Borroto R, Suarez J. Emergency Department patients with gastrointestinal symptoms during Federal holidays, Miami-Dade 2007 2010. Emerging Health Threats Journal 2011;4:13.

255. Borroto R, Suarez J, Rico E, O’Connell E, Llau A, Londono L, et al. Tracking emergency department (ED) patients with gastrointestinal symptoms during a Norovirus epidemic in Miami-Dade. Emerging Health Threats Journal 2011;4(1):s121.

256. Borroto R, Zhang G. Using ESSENCE to Identify a Seasonal Pattern of Gastrointestinal Syndrome in Miami-Dade. ISDS; Miami, Fl 2009.

257. Londoño L, O'Connell E, Zhang G, Leguen F. Utility of the Exposure Syndrome in ESSENCE. ISDS; Miami, Fl2009.

258. Cookson ST, Buehler JW, Lance SE. Georgia’s Strategy for Targeting Syndromic Surveillance. Advances in Disease Surveillance 2006;1:16.

259. Cameron W, Neu AL, Murray EL, Soetebier K, Cookson ST. Responding to Syndromic Surveillance Alerts: An Adaptable Protocol for Georgia Health Districts. Advances in Disease Surveillance 2007;2:95.

260. Finton RJ, Kolhe P, Katkowski SR, Cameron W, Murray EL. Event Detection in a Vulnerable Population. Advances in Disease Surveillance 2007;4:162.

261. Murray EL, Soetebier K, Cameron W. Syndromic Surveillance and Influenza-like Illness in Georgia. Advances in Disease Surveillance 2007;4:179.

262. Murray EL, Soetebier K, Neu AL, Witherspoon CG, Cookson ST. The Impact of Hurricane Katrina Evacuees from Louisiana and Mississippi on Georgia Emergency Departments: Syndromic Surveillance and Disasters. Advances in Disease Surveillance 2007;2:164.

263. Usman E, Harp S, Koppanyi Z. Late Season Influenza-Like Illness in Georgia: Prospective Detection of an Illness Cluster Using Emergency Department Syndromic Surveillance. Advances in Disease Surveillance 2007;2:170.

264. Gonzalez GA, Murray EL, Soetebier K, Buehler JW, Cookson ST. A Comparison between Syndromic Surveillance 911/EMS and Emergency Department Data. Advances in Disease Surveillance 2007;2:104.

265. Peace D, Smith W, Reeves A, Little D, Soetebier K, Drenzek C. A comparison of syndromic surveillance chief complaint data and discharge data in a pediatric hospital system during 2009 H1N1. Emerging Health Threats Journal 2011;4(1):s151.

266. Borroto R, Williamson B, Pitcher P, Ballester L, Smith W, Soetebier K, et al. Using Syndromic Surveillance Alert Protocols for Epidemiologic Response in Georgia. Online Journal of Public Health Informatics 2017; Vol 9, No 12017;9(1):e123.

267. Hatcher B, Cameron W, Soetebier K, Clarkson L, Cole D, Horan J. An Evaluation of the Georgia Syndromic Surveillance Program using CDC’s Guidelines for Evaluating Public Health Surveillance System. ISDS; Miami, Fl 2009.

268. Patel MT, Hoferka S. An Evaluation of Heat-Related Emergency. Online Journal of Public Health Informatics 2014;6(1):e88.

269. Brooks I, Vaid A, Edwards W. Analysis of 5 years of multistream surveillance and weather data in Champaign County. Emerging Health Threats Journal 2011;4:15.

270. Bemis K, Frias M, Patel MT, Christiansen D. Using an Emergency Department Syndromic Surveillance System to Evaluate Reporting of Potential Rabies Exposures, Illinois, 2013-2015. Public Health Reports. 2017;132(1).

271. Bemis K, Patel MT, Frias M, Christiansen D. Utility of Syndromic Surveillance in Detecting Potential Human Exposures to Rabies. Online Journal of Public Health Informatics 2016;8(1):e10.

272. Patel M, Adighibe E, Lombardo J, Loschen W, Stewart M, Vernon MO. Using Cloud Technology to Support Monitoring During High Profile Events. Online Journal of Public Health Informatics 2013;5(1):e73.

273. Bemis K, Gray S, Patel MT, Christiansen D. Disproportionate Emergency Room Use as an Indicator of Community Health. Online Journal of Public Health Informatics 2016;8(1):e9.

274. Silva JC, Rumoro DP, Shah SC, Gibbs GS, Hallock MM, Waddell M, et al. Adaptation of GUARDIAN for Syndromic Surveillance During the NATO Summit. Online Journal of Public Health Informatics 2013;5(1):e192.

275. Hafen RP, Anderson DE, Cleveland WS, Maciejewski R, Ebert DS, Abusalah A, et al. Syndromic surveillance: STL for modeling, visualizing, and monitoring disease counts. BMC Medical Informatics and Decision Making 2009;9:21.

276. Zou J, Karr AF, Datta G, Lynch J, Grannis S. A Bayesian spatio-temporal approach for real-time detection of disease outbreaks: A case study. BMC Medical Informatics and Decision Making 2014;14(1).

277. Hibler E. Evaluation of Pilot Hospitals Participating in the Indiana Public Health Emergency Surveillance System (PHESS). Advances in Disease Surveillance 2006;1:32.

278. Hibler E, Trepanier D, Wade M. Expanding the Functionality of Syndromic Surveillance Systems: Data Mining and Query Development. Advances in Disease Surveillance 2007;2:106.

279. Wade M, Gibson J, Dearth S, Grannis S. Operational Considerations and Early Successes with a Statewide Public Health Surveillance System. Advances in Disease Surveillance 2007;2:123.

280. Wade M, Trepanier D. Increasing Local Access to Syndromic Surveillance Data. Advances in Disease Surveillance 2007;4:203.

281. Maciejewski R, Rudolph S, Grannis SJ, Ebert DS. The Day-of-the-Week Effect: A Study Across the Indiana Public Health Emergency Surveillance System. Advances in Disease Surveillance 2008;5:44.

282. Dearth S, Gibson J. Increasing the Return-on-Investment from Syndromic Surveillance: Putting the Systems to Work for Routine Local Health Department Activities. Advances in Disease Surveillance 2007;4:158.

283. Wade M, Gorsuch S, Duszynski T, Gentry R. Exercise Demonstrates Effective Syndromic Surveillance Response Process. Advances in Disease Surveillance 2007;4:201.

284. Grannis S, Dixon B, Xia Y, Wu J. Using Information Entropy to Monitor Chief Complaint Characteristics and Quality. Online Journal of Public Health Informatics 2013;5(1):e16.

285. Fowler BE, Wade ME, Campbell RJ, DeFrancesco SJ, Grannis SJ, A.Trepanier D. Enhancing Syndromic Surveillance through Cross-border Data Sharing. Advances in Disease Surveillance 2008;5:31.

286. Dearth S, Mulanya K, Butwin J. Using a real-time syndromic surveillance system to track heat-related illnesses during a heat wave. Emerging Health Threats Journal 2011;4:39.

287. Carrico R, Goss L. Syndromic surveillance: Hospital emergency department participation during the Kentucky Derby Festival. Disaster Management and Response 2005;3(3):73-9.

288. Goss L, Carrico R, Hall C, Humbaugh K. A day at the races: Communitywide syndromic surveillance during the 2002 Kentucky Derby Festival. Journal of Urban Health 2003;80(1):i124-i.

289. Fleischauer AT, Young S, Mott J, Ratard R. Disaster Surveillance Revisited: Passive, Active and Electronic Syndromic Surveillance During Hurricane Katrina, New Orleans, LA – 2005. Advances in Disease Surveillance 2007;2:153.

290. Iberg Johnson J, Hand J, Ratard R. Near Real-Time Monitoring of Emergency Department Syndromic Surveillance Data During the 2013 Super Bowl and Mardi Gras Events in New Orleans, LA. Online Journal of Public Health Informatics 2014;6(1):e102.

291. Iberg Johnson J. Comparison of Three Critical Syndrome Classifications: Louisiana vs. BioSense. Online Journal of Public Health Informatics 2015;7(1):e30.

292. Iberg Johnson J, Brown K. Validation of Emergency Department and Outpatient Data Using ILI syndrome classifiers. Online Journal of Public Health Informatics 2015;7(1):e83.

293. Iberg Johnson J, Scott-Waldron C. What’s the Buzz About Arboviral Disease Syndromic Surveillance? Online Journal of Public Health Informatics 2016;8(1):e62.

294. Serrano J. Delay between Discharge and Admit Time Delay in ADT-A03 messages via LEEDS. Online Journal of Public Health Informatics 2017;9(1):e34.

295. Kirkwood A, Guenther E, Fleischauer AT, Gunn J, Hutwagner L, Barry MA. Direct cost associated with the development and implementation of a local syndromic surveillance system. Journal of Public Health Management & Practice 2007;13(2):194-9.

296. Centers for Disease Control Prevention. Norovirus activity--United States, 2006-2007. MMWR Morbidity and Mortality Weekly Report 2007;56(33):842-6.

297. Daniel JB, Heisey-Grove D, Gadam P, Yih W, Mandl K, DeMaria Jr A, et al. Connecting Health Departments and Providers: Syndromic Surveillance's Last Mile. MMWR Morbidity & Mortality Weekly Report 2005;54:147-50.

298. Gunn J, Pendarvis J, Barry A. Syndromic Surveillance and Zip Code Data: The Role of Zip Codes in Understanding Populations. Advances in Disease Surveillance 2006;1:29.

299. Gunn J, Pendarvis J, Smith AK, Barry MA. Do women really complain more then men? Looking at gastrointestinal chief complaints in Boston. Advances in Disease Surveillance 2006;1:28.

300. Pendarvis J, Gunn J, Smith AK, Barry A. Syndromic Surveillance in Boston: Validating the Detection of Small Events and Integrating Response from a Local Health Department. Advances in Disease Surveillance 2006;1:59.

301. Donovan M, Gunn JE. Using the Emergency Medical Text Processor to Standardize Chief Complaints in Boston’s Syndromic Surveillance System. Advances in Disease Surveillance 2007;4:49.

302. Ostrem M, Gunn JE. Syndromic Surveillance in Support of an Urban Violence Intervention. Advances in Disease Surveillance 2007;2:166.

303. Pendarvis J, Gunn J, Smith AK, Donovan M, Barry A. Sneezes vs. Wheezes: Syndrome Definitions for Influenza-like Illness. Advances in Disease Surveillance 2007;2:115.

304. Schlegelmilch J, Gunn J, Pendarvis J, Donovan M, Vinjé J, Widdowson M-A, et al. Bio-Surveillance and Enhanced Situational Awareness. Advances in Disease Surveillance 2007;4:191.

305. Pendarvis J, Miramontes R, Schlegelmilch J, Fleischauer AT, Gunn J, Hutwagner L, et al. Analysis of Syndrome Definitions for Gastrointestinal Illness with ICD9 Codes for Gastroenteritis during the 2006-07 Norovirus Season in Boston. Advances in Disease Surveillance 2007;4:263.

306. May L, Rennick M, Gustafson L, Gunn J. A Syndromic Approach to Emergency Department Surveillance for Skin and Soft Tissue Infections. Online Journal of Public Health Informatics 2013;5(1):e61.

307. Rennick M, Gunn J, Donovan M, Shah S, Barry MA, Burkom H, et al. Syndromic surveillance for bicycle-related injuries in Boston, 20072010. Emerging Health Threats Journal 2011;4:123.

308. Hoen A, Buckeridge D, Charland K, Mandl K, Brownstein J. Effect of expanded recommendations for pediatric seasonal influenza vaccination: an international comparison. Emerging Health Threats Journal 2011;4(1):s85.

309. Rennick M, Gunn J, Donovan M, Salvia J. Asthma patterns in Boston emergency department visits for children age five and under. Emerging Health Threats Journal 2011;4(1):s145.

310. Brown H, Romanosky A, Aslam S, Chu A, Blythe D. Early Identification of Salmonella Cases Using Syndromic Surveillance. Advances in Disease Surveillance 2008;5:94.

311. Bankoski A, Salim A, Faigen Z. Emergency Department Chief Complaint Versus Discharge Diagnosis for Tracking Disease Measures. Online Journal of Public Health Informatics 2014;6(1):e38.

312. Chu A, Brown H, Blythe D, Romanosky A. Can Syndromic Surveillance Data be Useful for Monitoring Respiratory Illness Activity? Advances in Disease Surveillance 2007;4:154.

313. Faigen Z, Salim A, Rojohn K, Isaac A, Adams S. Monitoring and auditing the transfer of syndromic surveillance data to ensure data completeness. Emerging Health Threats Journal 2011;4:52.

314. Salim A, Faigen Z, Ajit I, Adams S. Trends in emergency department visits for influenza-like illness and antiviral medication transactions. Emerging Health Threats Journal 2011;4:127.

315. Aslam S, Ajit I, Adams S, Faigen Z. Maryland ESSENCE expansion to incorporate prescription medication data. Emerging Health Threats Journal 2011;4(1):s61.

316. Aslam S, Ajit I, Adams S, Faigen Z. Utilities of Maryland’s syndromic surveillance system: indentifying threats, case investigation and situational awareness. Emerging Health Threats Journal 2011;4(1):s62.

317. Faigen Z, Ajit I, Aslam S, Adams S. Identification and tracking of heat-related illnesses using syndromic surveillance. Emerging Health Threats Journal 2011;4(1):s21.

318. Brown H, Aslam S, Ajit I, Adams S. Maryland ESSENCE Expansion to Achieve Statewide Coverage. ISDS; Miami, Fl 2009.

319. Brown H, Aslam S, Ajit I, Adams S. Syndromic Surveillance for ILI during H1N1 Response. ISDS; Miami, Fl 2009.

320. Mnatsakanyan ZR, Ashar RJ, Murphy SP, Coberly JS, Burkom H. Increase in Pneumonia Cases as an Early Indicator of Severe and Pandemic Influenza Outbreak. Advances in Disease Surveillance 2007;2:160.

321. Dambita N, Hirshon JM, Beilenson PL, Huffman J. The city of Baltimore’s multifaceted bioterrorism surveillance system. Journal of Urban Health 2003;80(1):i139-i.

322. DeVita S, Robbins A. Evaluating Usefulness of Maine's Syndromic Surveillance System for Hospitals, 2012. Online Journal of Public Health Informatics 2013;5(1):e174.

323. Rogers GM, Valenti A. Visual Portrayal of Syndromic Surveillance Data by Using High-Low Charting -- Maine, 2004. MMWR: Morbidity & Mortality Weekly Report 2005;54:207.

324. Sheline KD. Evaluation of the Michigan Emergency Department Syndromic Surveillance System. Advances in Disease Surveillance 2007;4:265.

325. Mamou F, Henderson T. Analysis of Heat Illness using Michigan Emergency Department Syndromic Surveillance. Online Journal of Public Health Informatics 2013;5(1):e139.

326. Swarnavel S, Collins J, Miller C. Validation of the Michigan’s Public Health Syndromic System Using Electronic Medical Records. Online Journal of Public Health Informatics 2015;7(1):e207.

327. Raman S, Levin J, Hall D, Frey K. Using Categorization of Reason-for-Visit Strings as the Basis for an Outbreak Detection System -- Minnesota, 2002-2003. MMWR: Morbidity & Mortality Weekly Report 2005;54:200.

328. Green K, Miller B, Hadidi M, Zimmerman M, Danila R. Dual-Model Approach to Syndromic Surveillance Using Hospital Emergency Department Data. MMWR: Morbidity & Mortality Weekly Report 2004;53:242.

329. Resch Z, Bullock C, Winslow A, Kelsey A. Missouri’s Syndromic Surveillance Experience. Advances in Disease Surveillance 2007;4:187.

330. Pugh KH, Kelsey A, Tominack R. Comparing Syndromic Surveillance and Poison Center Data for Snake Bites in Missouri. Online Journal of Public Health Informatics 2013;5(1):e102.

331. Wu F, Kelsey A. Early Detection of Influenza Activity Using Syndromic Surveillance in Missouri. Online Journal of Public Health Informatics 2013;5(1):e37.

332. Wu F, Braun C. Missouri Emergency Department Visits for Carbon Monoxide Poisoning. Online Journal of Public Health Informatics 2014;6(1):e50.

333. Kuo E, Wu F, Kelsey A. How Do Temperature, Relative Humidity and Heat Index Compare to Trends in Heat-Related Chief Complaints Captured by ESSENCE? ISDS; Miami, Fl 2009.

334. Wu F, Kuo E, Kelsey A. Utility of Syndromic Surveillance for Evaluating Influenza Trends. ISDS; Miami, Fl 2009.

335. Kuo E, Wu F, Kelsey A. A Comparison of Syndromic Surveillance and Mortality Statistics for Heat-Related Illness in Missouri. ISDS; Miami, Fl 2009.

336. Mohlenbrock WC, Rogers RF. Multi-Agency and Hospital, Syndromic Surveillance System in Montana. Advances in Disease Surveillance 2008;5:46.

337. Rappold AG, Stone SL, Cascio WE, Neas LM, Kilaru VJ, Carraway MS, et al. Peat bog wildfire smoke exposure in rural North Carolina is associated with cardiopulmonary emergency department visits assessed through syndromic surveillance. Environmental Health Perspectives. 2011;119(10):1415-20.

338. Harduar Morano L, Waller AE. Evaluation of the Components of the North Carolina Syndromic Surveillance System Heat Syndrome Case Definition. Public Health Reports. 2017;132(1):40S-7S.

339. Ising A, Proescholdbell S, Harmon KJ, Sachdeva N, Marshall SW, Waller AE. Use of syndromic surveillance data to monitor poisonings and drug overdoses in state and local public health agencies. Injury Prevention 2016;22:i43–i9.

340. Haas SW, Travers D, Waller A, Mahalingam D, Crouch J, Schwartz TA, et al. Emergency Medical Text Classifier: New system improves processing and classification of triage notes. Online Journal of Public Health Informatics. 2014;6(2):e178.

341. Modarai F, Mack K, Hicks P, Benoit S, Park S, Jones C, et al. Relationship of opioid prescription sales and overdoses, North Carolina. Drug and Alcohol Dependence 2013;132(1-2):81-6.

342. Sickbert-Bennett EE, Scholer M, Butler J, Travers D, MacFarquhar J, Waller A, et al. Evaluating a Syndromic Surveillance System for the Detection of Acute Infectious Gastroenteritis Outbreaks -- North Carolina, 2004. MMWR: Morbidity & Mortality Weekly Report 2005;54:201-.

343. Ising AI, Travers DA, MacFarquhar J, Kipp A, Waller AE. Triage Note in Emergency Department-Based Syndromic Surveillance. Advances in Disease Surveillance 2006;1:34.

344. Li M, Ising A, Waller AE, Falls D, Eubanks T, Kipp A. North Carolina Bioterrorism and Emerging Infection Prevention System. Advances in Disease Surveillance 2006;1:80.

345. MacFarquhar J, Sickbert-Bennett E, Waller A, Travers D, Scholer M, Davies M. Evolution of a Syndromic Surveillance Case Definition. Advances in Disease Surveillance 2006;1:46.

346. Travers D, Kipp A, MacFarquhar J, Waller A. Evaluation of Emergency Medical Text Processor For Pre-Processing Chief Complaint Data for Syndromic Surveillance. Advances in Disease Surveillance 2006;1:71.

347. Barnett C, Deyneka L, Waller A. Post-Katrina Situational Awareness in North Carolina. Advances in Disease Surveillance 2007;2:142.

348. Barnett C, Ising A, Travers D, Waller A. Emergency Department Data Quality Best Practices. Advances in Disease Surveillance 2007;2:193.

349. Falls DM, McLamb JR, Ising AI, Waller AE. Using Business Intelligence Tools to Automate Data Capture and Reporting. Advances in Disease Surveillance 2007;2:6.

350. Ising A, Travers D, Crouch J, Waller A. Improving Negation Processing in Triage Notes. Advances in Disease Surveillance 2007;4:50.

351. Li M, Ising A, Havaldar R, Waller A. Multi-Tier Role Based Access for Secure and Flexible Syndromic Surveillance. Advances in Disease Surveillance 2007;2:158.

352. Li M, Ising A, Deyneka L, Falls D, Waller A. Using NC DETECT Summary Reports to Share Syndromic Information. Advances in Disease Surveillance 2008;5:113.

353. Ratcliffe A, Barnett C, Ising A, Waller A. Evaluating the Validity of ED Visit Data for Biosurveillance. Advances in Disease Surveillance 2008;5:57.

354. Burkom H, Burrer S, Barker L, Robison V, Hicks P, Ising A. Use of Syndromic Data to Determine Oral Health Visit Burden on Emergency Departments. Online Journal of Public Health Informatics 2013;5(1):e57.

355. Deyneka L, Ising A, Li M. Enhanced Surveillance during the Democratic National Convention, Charlotte, NC. Online Journal of Public Health Informatics 2013;5(1):e190.

356. Harmon KJ, Proescholdbell S, Marshall S, Waller A. Utilization of Emergency Department Data for Drug Overdose Surveillance in North Carolina. Online Journal of Public Health Informatics 2014;6(1):e174.

357. Ising A, Barnett C, Falls D, Waller AE, Wallace J, Deyneka L. Assessing the Potential Impact of the BioSense 24-hour Rule Using NC DETECT ED Data. Online Journal of Public Health Informatics 2015;7(1):e84.

358. Faigen Z, Ising A, Deyneka L, Waller AE. Triage Notes in Syndromic Surveillance – A Double Edged Sword. Online Journal of Public Health Informatics 2016;8(1):e179.

359. Deyneka L, Costa P, Kipp A. Using Poison Center Syndromic Surveillance for Environmental Health Signals Detection. Advances in Disease Surveillance 2007;2:151.

360. Ising A, Li M, Deyneka L, Barnett C, Scholer M, Waller A. Situational Awareness Using Web-based Annotation and Custom Reporting. Advances in Disease Surveillance 2007;4:167.

361. Brannan S, Evens N, Barnett C, Deyneka L, Ising A, Wheaton B, et al. Web-Based Spatio-Temporal Display of NC DETECT Surveillance Data. Advances in Disease Surveillance 2008;5:6.

362. Ising A, Li M, Deyneka L, Vaughan-Batten H, Waller A. Improving syndromic surveillance for nonpower users: NC DETECT dashboards. Emerging Health Threats Journal 2011;4:75.

363. Rhea S, Ising A, Waller A, Deyneka L, Vaughan-Batten H, Haskell MG. Animal bite surveillance using NC DETECT. Emerging Health Threats Journal 2011;4:124.

364. Travers D, Lich KH, Lippmann S, Waller A, Weinberger M, Yeatts K. Defining emergency department asthma visits for public health surveillance. Emerging Health Threats Journal 2011;4:152.

365. Vaughan-Batten H, Deyneka L, Ising A, Waller A. Data requests for research: best practices based on the North Carolina DETECT experience. Emerging Health Threats Journal 2011;4:153.

366. Lamb E, Vaughan-Batten H, Dailey N, Maillard J-M, Johns L, Fleischauer A, et al. Emergency department diagnosis code data for surveillance of vaccine adverse events: comparison with the national vaccine adverse event reporting system. Emerging Health Threats Journal 2011;4(1):s90.

367. Ising A, Rhea S, Deyneka L, Vaughan-Batten H. The use of NC DETECT ED data to examine heat-related illness. Emerging Health Threats Journal 2011;4(1):s30.

368. King R, Deyneka L, Vaughan-Batten H, Barker L, Robison V, Benoit S, et al. Use of syndromic surveillance systems for oral health surveillance. Emerging Health Threats Journal 2011;4(1):s32.

369. Falls D, Li M, Waller A. Securing protected health information in NC DETECT. Emerging Health Threats Journal 2011;4(1):s79.

370. Li M, Loschen W, Deyneka L, Burkom H, Ising A, Waller A. Time of Arrival Analysis in NC DETECT to Find Clusters of Interest from Unclassified Patient Visit Records. Online Journal of Public Health Informatics 2013;5(1):e13.

371. Young A, Fliss MD, Ising A. Improving Local Non-Communicable Disease Surveillance within a Changing Data Environment. Online Journal of Public Health Informatics 2015;7(1):e177.

372. Deyneka L, Hakenewerth A, Faigen Z, Ising A, Barnett C. Using Syndromic Surveillance Data to Monitor Endocarditis and Sepsis among Drug Users. Online Journal of Public Health Informatics 2017;9(1):e124.

373. Waller AE, Lippmann S, Ising A, Crump C. Childhood Injury in Wake County, NC: Local Use of Public Health Surveillance Data. Online Journal of Public Health Informatics 2015;7(1):e172.

374. Samoff E, Fleischauer A, Moore Z, Park M, Deyneka L, Davis M, et al. Use of Syndromic Surveillance for Outbreak Detection and Management, North Carolina 2008-9. ISDS; Miami, Fl 2009.

375. Rao S, Johnson A, Abbott D, Frederick J, Caram LB, Woods C. Clinical Surveillance Markers of Influenza-like Illness (ILI) at a Veterans Affairs (VA) Hospital. Advances in Disease Surveillance 2007;4:186.

376. Musumba A, Feist M, Kubischta L, Miller T, Goplin J. Evaluation of ILI Data Use to Augment Traditional Influenza Surveillance. Advances in Disease Surveillance 2008;5:120.

377. Goplin J, Feist M, Miller T. Variation of Chief Complaint-Based Respiratory Symptom Data in One Hospital’s Nurse Advice Call Center and Emergency Department. Advances in Disease Surveillance 2007;2:105.

378. Gonzalez S, Newmyer A, Qu M. Validation of SyS Data to Inform Surveillance of Health Disparities in Nebraska. Online Journal of Public Health Informatics 2016;8(1):e59.

379. Murphy E, Safranek T. Assessing the use of syndromic surveillance data to identify and track heat illness in Nebraska, 20102011. Emerging Health Threats Journal 2011;4:105.

380. Gonzlez S, DeVries D, Qu M. Use of Near-Real–Time Data to Inform Underage Drinking Surveillance in Nebraska. Online Journal of Public Health Informatics 2017;9(1):e142.

381. Daly ER, Dufault K, Swenson DJ, Lakevicius P, Metcalf E, Chan BP. Use of Emergency Department Data to Monitor and Respond to an Increase in Opioid Overdoses in New Hampshire, 2011-2015. Public Health Reports. 2017;132(1).

382. Swenson D, Adamski C, Armenti K. Real-Time Surveillance: Cutting-Edge Technology in NH. Advances in Disease Surveillance 2007;2:29.

383. Swenson DJ, Taylor C, Southworth J, Dufault K. Surveillance Investigation Tool Development Targeted For Results. Advances in Disease Surveillance 2007;4:115.

384. Miller S, Fallon K, Anderson L. New Hampshire emergency department syndromic surveillance system. Journal of Urban Health 2003;80(1):i118-i.

385. Swenson D, Zhang X, Miller S, Dufault K, Taylor C, Fallon K, et al. Ten years of syndromic surveillance in New Hampshire: innovation, experience and outcomes. Emerging Health Threats Journal 2011;4:148.

386. Swenson D, Mayo L, Dufault K, Daly E, Bascom S, Dionne-Odom J, et al. Use of emergency department data for case finding following a community anthrax exposure. Emerging Health Threats Journal 2011;4(1):s118.

387. Borjan M, Lumia M. Evaluation of a state based syndromic surveillance system for the classification and capture of non-fatal occupational injuries and illnesses in New Jersey. American Journal of Industrial Medicine. 2017;60(7):621-6.

388. Tsai S, Hamby T, Chu A, Gleason JA, Goodrow GM, Gu H, et al. Development and Application of Syndromic Surveillance for Severe Weather Events Following Hurricane Sandy. Disaster Medicine and Public Health Preparedness. 2016;10(3):463-71.

389. Berry M, Fagliano J, Tsai S, McGreevy K, Walsh A, Hamby T. Evaluation of Heat-related Illness Surveillance Based on Chief Complaint Data from New Jersey Hospital Emergency Rooms. Online Journal of Public Health Informatics 2013;5(1):e125.

390. Hamby T, Tsai S, Genese C, Walsh A, Bradford L, Lifshitz E. Paralysis Analysis: Investigating Paralysis Visit Anomalies in New Jersey. Online Journal of Public Health Informatics 2013;5(1):e126.

391. Hamby T, Pomary V, Adler E, Tsai S, Walsh A. School Daze: Capturing Chemical Exposures in Syndromic Surveillance - New Jersey 2013. Online Journal of Public Health Informatics 2014;6(1):e64.

392. Chen F, Kostial E, Hamby T, Tsai S. Validation of New Jersey Emergency Department (ED) Registration Data in BioSense 2.0. Online Journal of Public Health Informatics 2015;7(1):e118.

393. Chu AF, Tsai S, Hamby T, Kostial E, Fagliano J. Development of Mental Health Classification Related to Severe Weather Events. Online Journal of Public Health Informatics 2015;7(1):e120.

394. Erdogdu P, Hamby T, Tsai S. Better, Stronger, Faster: Why Add Fields to Syndromic Surveillance? New Jersey, 2015. Online Journal of Public Health Informatics 2016;8(1):e106.

395. Hamby T, Tsai S, McHugh L. Surveillance in New Jersey*from anthrax to automation. Emerging Health Threats Journal 2011;4:70.

396. Borjan M, Lumia M. Classification and Capture of Work-Related Non-Fatal Injuries Through a Real-Time Syndromic Surveillance System. Online Journal of Public Health Informatics 2016;8(1):e94.

397. Hamby T, Tsai S, Gu H. How’s the Weather? Severe Weather Classifications in Syndromic Surveillance. Online Journal of Public Health Informatics 2017;9(1):e30.

398. Erdogdu P, Tsai S, Hamby T. HAI Surveillance Enhancement within EpiCenter by Utilization of Triage Notes. Online Journal of Public Health Informatics 2017;9(1):e40.

399. Hamby T, Tsai S, Gu H, Goodrow G, Gleason J, Fagliano J. Weather Outlook: Cloudy with a Chance of...— Classification of Storm-Related ED Visits. Online Journal of Public Health Informatics 2016;8(1):e118.

400. La Forgia B, Fiorenza L, John S, Paladini M. Daily Electronic Disease Surveillance System -- Bergen County, Paramus, New Jersey. MMWR: Morbidity & Mortality Weekly Report 2005;54:192-.

401. Paladini M. Daily Emergency Department Surveillance System — Bergen County, New Jersey. MMWR: Morbidity & Mortality Weekly Report 2004;53:47-9.

402. Brillman JC, Burr T, Forslund D, Joyce E, Picard R, Umland E. Modeling emergency department visit patterns for infectious disease complaints: results and application to disease surveillance. BMC Medical Informatics and Decision Making 2005;5:4.

403. Lauper U, Chen J-H, Lin S. Window of Opportunity for New Disease Surveillance: Developing Keyword Lists for Monitoring Mental Health and Injury Through Syndromic Surveillance. Disaster Medicine and Public Health Preparedness. 2017;11(2):173-8.

404. Chang H-G, Cochrane DG, Tserenpuntsag B, Allegra JR, Smith PF. ICD9 as a Surrogate for Chart Review in the Validation of a Chief Complaint Syndromic Surveillance System. Advances in Disease Surveillance 2006;1:11.

405. Thoburn KK, Miller JR, Chen J-H, Schmit KJ, Chang H-G. The New York State Department of Health’s Syndromic Surveillance System. Advances in Disease Surveillance 2006;1:68.

406. Chen J, Schmit K, Chang H, Miller J. Detection of Carbon Monoxide Poisoning in Chief Complaint Data. Advances in Disease Surveillance 2007;4:153.

407. Cochrane DG, Allegra JR, McCarthy C, Luk J, Chang H-G, Chen J-H. The Utility of Patient Chief Complaint and ICD 9 Classifiers for the Influenza Sub-syndrome. Advances in Disease Surveillance 2008;5:15.

408. Chang H-G, Weng C, DiDonato C, DiCesare D, Chen J-H, Blog D. Evaluation of Emergency Department Data Quality following PHIN Syndromic Surveillance Messaging Guide. Online Journal of Public Health Informatics 2013;5(1):e50.

409. Chen J-H, Lauper U, Pantea C, Lin S, Chang H-G. Carbon Monoxide Poisoning during Hurricane Sandy in Affected New York State Counties. Online Journal of Public Health Informatics 2015;7(1):e119.

410. Chang H-G, Chen J-H, Cochrane DG, Allegra JR, Burkom H, Tokars JI, et al. A Pilot Study of Aberration Detection Algorithms with Simulated Data. Advances in Disease Surveillance 2007;4:240.

411. Weng C, Chen J-H, DiDonato C, Chang H-G. Map application to the New York state electronic syndromic surveillance system. Emerging Health Threats Journal 2011;4(1):s156.

412. Terry W, Ostrowsky B, Huang A. Should We Be Worried? Investigation of Signals Generated by an Electronic Syndromic Surveillance System -- Westchester County, New York. MMWR: Morbidity & Mortality Weekly Report 2004;53:190-5.

413. Cho SS, Hackmyer S, Li J, Lipsman J, Meruelo O, Mottola D, et al. Establishing an Automated Surveillance System. MMWR: Morbidity & Mortality Weekly Report 2004;53:232.

414. Mathes RW, Lall R, Levin-Rector A, Sell J, Paladini M, Konty KJ, et al. Evaluating and implementing temporal, spatial, and spatio-temporal methods for outbreak detection in a local syndromic surveillance system. PLoS ONE 2017;12(9):e0184419.

415. Mathes RW, Ito K, Lane K, Matte TD. Real-time surveillance of heat-related morbidity: relation to excess mortality associated with extreme heat. PLoS ONE 2017;12(9):e0184364.

416. Kotzen M, Sell J, Mathes RW, Dentinger C, Lee L, Schiff C, et al. Using syndromic surveillance to investigate tattoo-related skin infections in New York City. PLoS ONE 2015;10(6):e0130468.

417. Bregman B, Slavinski S. Using emergency department data to conduct dog and animal bite surveillance in New York City, 2003-2006. Public Health Reports 2012;127(2):195-201.

418. Plagianos MG, Wu WY, McCullough C, Paladini M, Lurio J, Buck MD, et al. Syndromic surveillance during pandemic (H1N1) 2009 outbreak, New York, New York, USA. Emerging Infectious Diseases 2011;17(9):1724-6.

419. Marx MA, Rodriguez CV, Greenko J, Das D, Heffernan R, Karpati AM, et al. Diarrheal illness detected through syndromic surveillance after a massive power outage: New York City, August 2003. American Journal of Public Health 2006;96(3):547-53.

420. Heffernan R, Mostashari F, Das D, Karpati A, Kulldorff M, Weiss D. Syndromic surveillance in public health practice, New York City. Emerging Infectious Diseases 2004;10(5):858-64.

421. Das D, Weiss D, Mostashari F, Treadwell T, McQuiston J, Hutwagner L, et al. Enhanced drop-in syndromic surveillance in New York City following September 11, 2001. Journal of Urban Health 2003;80:i76-i88.

422. Lall R, Abdelnabi J, Ngai S, Parton HB, Saunders K, Sell J, et al. Advancing the Use of Emergency Department Syndromic Surveillance Data, New York City, 2012-2016. Public Health Reports 2017;132(1).

423. Nolan ML, Kunins HV, Lall R, Paone D. Developing Syndromic Surveillance to Monitor and Respond to Adverse Health Events Related to Psychoactive Substance Use: Methods and Applications. Public Health Reports 2017;132(1).

424. Wilson EL, Egger JR, Konty KJ, Paladini M, Weiss D, Trang QN. Description of a School Nurse Visit Syndromic Surveillance System and Comparison to Emergency Department Visits, New York City. American Journal of Public Health 2014;104(1).

425. Wallace DJ, Arquilla B, Heffernan R, Kramer M, Anderson T, Bernstein D, et al. A test of syndromic surveillance using a severe acute respiratory syndrome model. American Journal of Emergency Medicine 2009;27(4):419-23.

426. J Ackelsberg, S Balter, K Bornschelgel, E Carubis, B Cherry, V D Das, et al. Syndromic surveillance for bioterrorism following the attacks on the World Trade Center -- New York City, 2001. MMWR: Morbidity & Mortality Weekly Report 2002;51:13-5.

427. Hsieh JL, Nguyen TQ, Matte T, Ito K. Drinking water turbidity and emergency department visits for gastrointestinal illness in New York City, 2002-2009. PLoS ONE 2015;10(4).

428. Westheimer E, Paladini M, Balter S, Weiss D, Fine A, Nguyen TQ. Evaluating the New York City emergency department syndromic surveillance for monitoring influenza activity during the 2009-10 influenza season. PLoS Currents 2012:1-15.

429. Mathes RW, Ito K, Matte T. Assessing syndromic surveillance of cardiovascular outcomes from emergency department chief complaint data in New York City. PLoS ONE 2011;6(2).

430. Beatty ME, Phelps S, Rohner C, Weisfuse I. Blackout of 2003: Public Health Effects and Emergency Response. Public Health Reports 2006;121(1):36-44.

431. Seil K, Marcum J, Lall R, Stayton C. Utility of a near real-time emergency department syndromic surveillance system to track injuries in New York City. Injury Epidemiology 2015;2(1):11.

432. Minen MT, Boubour A, Wahnich A, Grudzen C, Friedman BW. A Retrospective Nested Cohort Study of Emergency Department Revisits for Migraine in New York City. Headache 2018;58(3):399-406.

433. Chen BC, Shawn LK, Connors NJ, Wheeler K, Williams N, Hoffman RS, et al. Carbon monoxide exposures in New York City following Hurricane Sandy in 2012. Clinical Toxicology 2013;51(9):879-85.

434. Heffernan R, Mostashari F, Das D, Besculides M, Rodriguez C, Greenko J, et al. New York City Syndromic Surveillance Systems. MMWR: Morbidity & Mortality Weekly Report 2004;53:25-7.

435. Metzger KB, Hajat A, Crawford M, Mostashari F. How Many Illnesses Does One Emergency Department Visit Represent? Using a Population-Based Telephone Survey To Estimate the Syndromic Multiplier. MMWR: Morbidity & Mortality Weekly Report 2004;53:106-11.

436. Steiner-Sichel L, Greenko J, Heffernan R, Layton M, Weiss D. Field Investigations of Emergency Department Syndromic Surveillance Signals -- New York City. MMWR: Morbidity & Mortality Weekly Report 2004;53:184-9.

437. Balter S, Weiss D, Hanson H, Reddy V, Das D, Heffernan R. Three Years of Emergency Department Gastrointestinal Syndromic Surveillance in New York City: What Have we Found? MMWR: Morbidity & Mortality Weekly Report 2005;54:175-80.

438. Goranson C, Konty K, Lu J, Mostashari F. Visualization of Syndromic Surveillance Using GIS. Advances in Disease Surveillance 2006;1:26.

439. Das D, Olson D, Heffernan R. Estimating Hospital Admissions for Influenza Using Emergency Department (ED) Syndromic Surveillance Data, New York City. Advances in Disease Surveillance 2007;2:150.

440. Murray EL, Heffernan R, Yeung A, Kidoguchi LS, Weiss D. Use of Syndromic Surveillance in the Investigation of Salmonella wandsworth Outbreak. Advances in Disease Surveillance 2007;4:180.

441. Lu J, Metzger K, Cajigal A, Konty K, Matte T. Identifying and Modeling Spatial Patterns of Heat-Related Illness in New York City. Advances in Disease Surveillance 2007;4:255.

442. Cajigal A, Heller D, Paone D, Konty K. Monitoring Spatial Patterns of Adverse Drug Events and Morbidity in New York City Using Syndromic Data Streams. Advances in Disease Surveillance 2008;5:168.

443. Goranson C, K T, T T, Cajigal A, Paladini M, Murray EL, et al. Cluster Detection Comparison in Syndromic Surveillance. Advances in Disease Surveillance 2008;5:33.

444. Murray EL. Using Emergency Department Disposition Data to Monitor Hospitalizations for Influenza-like Illness. Advances in Disease Surveillance 2008;5:183.

445. Wilson EL, Murray EL, Jones L, Paladini M, Nguyen TQ. A Comparison of Electronic Emergency Department Visits and Data Collected Manually During a Field Exercise. Advances in Disease Surveillance 2008;5:136.

446. Jasek JP, Hosseinipour N, Rubin T, Lall R. Using Syndromic Emergency Department Data to Augment Oral Health Surveillance. Online Journal of Public Health Informatics 2013;5(1):e112.

447. Lall R, Paladini M. Evaluating Syndromic Data for Surveillance of Non-infectious Disease. Online Journal of Public Health Informatics 2013;5(1):e163.

448. Lane K, Lall R, Wheeler K, Ito K, Matte T. Evaluating Utility of Cold-Injury Syndromic Surveillance Data in New York City. Online Journal of Public Health Informatics 2013;5(1):e122.

449. Mathes R, Ito K, Matte T. Risk of Cardiovascular Morbidity and Mortality in Relation to Temperature. Online Journal of Public Health Informatics 2013;5(1):e144.

450. Mathes R, Metzger KB, Ito K, Matte T. Surveillance of Heat-related Morbidity: Relation to Heat-related Excess Mortality. Online Journal of Public Health Informatics 2013;5(1):e156.

451. Paladini M, Lall R, Schachterle SE. Differentiating ZIP Codes in Syndromic Data; What Can They Tell Us? Online Journal of Public Health Informatics 2013;5(1):e127.

452. Sell J, Mathes R, Paladini M. Detecting Changes in Chief Complaint Word Count: Effects on Syndromic Surveillance. Online Journal of Public Health Informatics 2013;5(1):e21.

453. Sell J, Wong A. A Survey of Data Recording Procedures at New York City Emergency Departments. Online Journal of Public Health Informatics 2013;5(1):e114.

454. Lall R, Levin-Rector A, Mathes R, Weiss D. Detecting Unanticipated Increases in Emergency Department Chief Complaint Keywords. Online Journal of Public Health Informatics 2014;6(1):e93.

455. Agarwal M, Idaikkadar N, Weiss D. Epidemiology of Gunshot-Related Injuries in NYC Emergency Departments from 2004-2014. Online Journal of Public Health Informatics 2015;7(1):e7.

456. Chase AZ. Monitoring Trends of Self-diagnosis in New York City Emergency Departments. Online Journal of Public Health Informatics 2015;7(1):e186.

457. Chase AZ, Agarwal M, Mercurio-Zappala M, Su M. Using Syndromic Surveillance to Characterize Unintentional Ingestions in Children. Online Journal of Public Health Informatics 2015;7(1):e116.

458. Mathes R, Sell J, Tam AW, Levin-Rector A, Lall R. Building a Better Syndromic Surveillance System: the New York City Experience. Online Journal of Public Health Informatics 2015;7(1):e39.

459. Tam AW, Toprani A, Mathes R. Reciprocal Data Sharing: Sending Monthly Summary Reports of Syndromic Data to ED. Online Journal of Public Health Informatics 2015;7(1):e164.

460. Lu J, Konty K, Goranson C, Mostashari F. Algorithms to Characterize Syndromic Surveillance Spatial Alerts. Advances in Disease Surveillance 2007;2:56.

461. Cajigal A, Konty K, Heller D. Using a syndromic approach to monitor alcohol-related visits of college-aged emergency department patients. Emerging Health Threats Journal 2011;4:22.

462. Konty K, Olson D. The spatial-temporal pattern of excess influenza visits at the (sub-)urban scale. Emerging Health Threats Journal 2011;4(1):**s35**.

463. Ngai S, Edelstein Z, Myers J, Weiss D. Tracking HIV Post-Exposure Prophylaxis using Syndromic Surveillance in NYC Emergency Departments. Online Journal of Public Health Informatics 2015;7(1):e89.

464. Westheimer E, Nguyen T, Paladini M, Weiss D, Balter S. Comparison of respiratory, febrile and influenza-like illness syndromes to detect laboratory-reported H1N1 and RSV, Influenza Season 2009–10, New York City. Emerging Health Threats Journal 2011;4(1):s105.

465. Yung J, Nkwocha P, Tam AW, Lall R, Mathes R. Comparison between HL7 and Legacy Syndromic Surveillance Data in New York City. Online Journal of Public Health Informatics 2015;7(1):e179.

466. Wahnich A, Lall R, Weiss D. Monitoring for Local Transmission of Zika Virus using Emergency Department Data. Online Journal of Public Health Informatics 2017;9(1):e115.

467. Olson D, van der Mei W, Lim S, Yoon C, Kull M, Davila M. Monitoring child mental health related emergency department visits in New York City. Online Journal of Public Health Informatics 2017;9(1):e134.

468. Sell J. In Denial: Symptom Negation in New York City Emergency Department Chief Complaints. Online Journal of Public Health Informatics 2017;9(1):e70.

469. Kotzen M, Mathes R, Lee L, Weiss D. Using Syndromic Surveillance to Investigate Tattoo-related Skin Infections in NYC. Online Journal of Public Health Informatics 2014;6(1):e172.

470. Athens J. Can We Use Syndromic Surveillance Data to Identify Primary Care Visits to NYC EDs? Online Journal of Public Health Informatics 2013;5(1):e56.

471. Mathes RW, Matte TD, Ito K. Assessing Syndromic Surveillance of Cardiovascular Outcomes from Emergency Department Chief Complaint Data in New York City. ISDS; Miami, Fl 2009.

472. Racer CF. Novel H1N1 Flu, Type A: the Media, and Syndromic Surveillance, April-July 2009. ISDS; Miami, Fl 2009.

473. Nguyen TQ, Paladini M, Balter S, Thorpe L. Using Emergency Department Syndromic Surveillance Disposition Data to Estimate Influenza-related Severity during Novel H1N1 Outbreak, 2009 — New York City. ISDS; Miami, Fl 2009.

474. Das D, Mostashari F, Weiss D, Balter S, Heffernan R. Monitoring Over-the-Counter Pharmacy Sales for Early Outbreak Detection — New York City, August 2001–September 2003. MMWR: Morbidity & Mortality Weekly Report 2004;53:235.

475. Marx MA, Rodriguez C, Greenko J, Das D, Mostashari F, Balter S, et al. Investigation of Diarrheal Illness Detected Through Syndromic Surveillance After a Massive Blackout — New York City, August 2003. MMWR: Morbidity & Mortality Weekly Report 2004;53:251.

476. Gallagher T. Open Source Development of Syndromic Investigation Decision Support Tools. Advances in Disease Surveillance 2008;5:104.

477. Thomas RM, Fowler BE. Monitoring Hospital Emergency Department Visits for Chief Complaints of Insect Bites after a Major Flood. Advances in Disease Surveillance 2008;5:193.

478. Brown AL, Storm WE, Fowler BE. Tracking Drug Overdose Trends in Ohio using ED Chief Complaints. Online Journal of Public Health Informatics 2013;5(1):e121.

479. Brown A, Storm W, Fowler B. Using syndromic surveillance data to identify emerging trends in designer drug use. Emerging Health Threats Journal 2011;4:16.

480. Gallagher T. Enhancing state biosurveillance: situational awareness via local context analysis. Emerging Health Threats Journal 2011;4:63.

481. Storm W, Bennett B, Fowler B. Using chief complaint data to evaluate the effectiveness of a statewide smoking ban. Emerging Health Threats Journal 2011;4(1):s152.

482. Orr C, VanBuskirk K. Utilization of the Syndromic Surveillance Framework for Detection of Heat-Related illnesses. Advances in Disease Surveillance 2007;4:60.

483. Hines JZ, Bancroft J, Powell M, Hedberg K. Case Finding Using Syndromic Surveillance Data During an Outbreak of Shiga Toxin--Producing Escherichia coli O26 Infections, Oregon, 2015. Public Health Reports 2017;132(4):448-50.

484. Powell M, Ryff K. Marketing a syndromic surveillance system to hospital emergency departments. Emerging Health Threats Journal 2011;4:119.

485. Powell M, Ryff K, Giffin S. Surveillance of poison center data using the National Poison Data System web service. Emerging Health Threats Journal 2011;4:120.

486. Storm B, Fowler B. Evaluating the relationship between heat-related ED visits and weather variables. Emerging Health Threats Journal 2011;4:146.

487. Powell M, Boyd L. Tracking Health Effects of Wildfires: The Oregon ESSENCE Wildfire Pilot Project. Online Journal of Public Health Informatics 2017;9(1):e141.

488. Jagger MA, Jaramillo S, Boyd L, Johnson B, Reed KR, Powell M. Mass Gathering Surveillance: New ESSENCE Report and Collaboration Win Gold in OR. Online Journal of Public Health Informatics 2017;9(1):e133.

489. Chapman WW, Christensen LM, Wagner MM, Haug PJ, Ivanov O, Dowling JN, et al. Classifying free-text triage chief complaints into syndromic categories with natural language processing. Artificial Intelligence in Medicine 2005;33(1):31-40.

490. Tsui F-C, Su H, Dowling J, Voorhees R, Espino J, Wagner M. An automated influenza-like-illness reporting system using freetext emergency department reports. Emerging Health Threats Journal 2011;4(1):s117.

491. Gevitz K, Madera R, Newbern C, Lojo J, Johnson CC. Risk of Fall-Related Injury due to Adverse Weather Events, Philadelphia, Pennsylvania, 2006-2011. Public Health Reports 2017;132(1).

492. Williams KA, Buechner JS. Evaluation of the Rhode Island Real-time Outbreak and Disease Surveillance (RI RODS) System: Disparate Data. Advances in Disease Surveillance 2007;2:219.

493. Lozier M, Martin C, Chaput D. Surveillance of Overdose-related Emergency Department Visits in Rhode Island. Online Journal of Public Health Informatics 2014;6(1):e165.

494. Williams KA, Espino J, Buechner JS. Syndromic Surveillance System User Satisfaction and Attitudes. Advances in Disease Surveillance 2007;2:220.

495. Cheng YE, Dhotre H, Das D, Drociuk D. Automatic and Secure Data Transfer of Syndromic Data between Hospitals and Public Health Using the PHINMS. Advances in Disease Surveillance 2008;5:14.

496. Dhotre H, Das D, Cheng E, Drociuk D. A Closer Look at Developing a Statewide Emergency Department Syndromic Surveillance System in a Rural State. Advances in Disease Surveillance 2008;5:24.

497. Johnson K, Alianell A, Radcliffe R. Seasonal Patterns in Syndromic Surveillance Emergency Department Data due to Respiratory Illnesses. Online Journal of Public Health Informatics 2014;6(1):e66.

498. Dhotre H, Springs C, Drociuk D, Cheng Y. A comparison of the fever–flu syndrome category with the SC ILINet surveillance system in South Carolina: 2009–2010 influenza season. Emerging Health Threats Journal 2011;4(1):s127.

499. Cheng Y, Dhotre H, Drociuk D. PHIN-MS deployment acceptability survey for the data transfer of syndromic data between hospitals and public health. Emerging Health Threats Journal 2011;4(1):s124.

500. Boeker S, Drociuk D, Jankelevich S, Kelly W, Steed C. Mutual Benefits in Partnerships between Hospital-based and Public Health Department Syndromic Surveillance Systems in Outbreak Detection and Investigation. Advances in Disease Surveillance 2007;4:146.

501. Boeker S, Drociuk D, Belflower AE, Steed C. A Novel Approach to Using Chief Complaint-Driven Syndromic Surveillance: Use of CDC's EARS-X by Hospital Infection Control Practitioners. Advances in Disease Surveillance 2007;2(144):144.

502. Lawson BM, Fitzhugh EC, Hall SP, Franklin C, Hutwagner LC, Seeman GM, et al. Multifaceted syndromic surveillance in a public health department using the Early Aberration Reporting System. Journal of Public Health Management & Practice 2005;11(4):274-81.

503. Chung WM, Buseman CM, Joyner SN, Hughes SM, Luby JP, Haley RW, et al. The 2012 West Nile encephalitis epidemic in Dallas, Texas. Journal of the American Medical Association 2013;310(3):297-307.

504. Adeleye O, Carvalho M, Halm M, Eshofonie A, Jr. ACR, AwosikaOlumo D. Using Syndromic Data to Investigate Gastrointestinal Illness Associated with Water Quality Complaints in Houston. Advances in Disease Surveillance 2007;4:141.

505. Halm M, Carvalho M, Eshofonie A, Awosika-Olumo D, Arafat R. Surveillance Zones: Geographic Aggregation of Syndromic Data for Improved Response. Advances in Disease Surveillance 2007;4:96.

506. Yang B, Wang N, McNeely W, Khuwaja S, Arafat R. Association between Influenza-like Illnesses and Social Determinants of Health by Census Tract in Houston/Harris County. Online Journal of Public Health Informatics 2014;6(1):e17.

507. Awosika-Olumo D, Mgbere O, Khuwaja S, Adeleye O, Arafat R. Relationship between Emergency Room (ER) Syndromic surveillance data and Influenza-Like Illness (ILI) Surveillance in Houston, Texas. Advances in Disease Surveillance 2007;4:145.

508. Stewart A, Carvalho M, Awosika-Olumo D. Mental Health Emergency Department Visits in Houston: Developing a Post-Disaster Mental Health Surveillance System. Advances in Disease Surveillance 2008;5:66.

509. Arnold RM, McNeely W, Muhetaer K, Yang B, Arafat RR. Application of Syndromic Surveillance to Describe Gunshot-related Injuries in Houston. Online Journal of Public Health Informatics 2015;7(1):e106.

510. Henry J, Murphy P, Pichette J, Andersen D, Cooks-Sinclair H. Natural disasters and use of syndromic surveillance: Austin, Texas Metro Area 2011. Emerging Health Threats Journal 2011;4:72.

511. Guerrero AC, Shim T, Kemple S, Nancy D. Walea, Viera F, Grota P, et al. Norovirus Outbreak Detected by Emergency Department Syndromic Surveillance using RedBat®. Advances in Disease Surveillance 2007;4:165.

512. Robertson J, Hill M. Evaluating the use of syndromic surveillance for the detection of influenza-like illness in Salt Lake County, Utah. Emerging Health Threats Journal 2011;4(1):s146.

513. Austin EE, Yang J, Powell T. Using GI Syndrome Data as an Early Warning Tool for Norovirus Outbreak Activity. Online Journal of Public Health Informatics 2013;5(1):e69.

514. Austin EE. Using Syndromic Surveillance Data to Describe Chronic High Frequency ED Utilization. Online Journal of Public Health Informatics 2015;7(1):e64.

515. Wahnich A, Hobron K, Austin EE, Powell T. Unintentional Drug Overdoses in Virginia: Analysis of Syndromic and Death Data. Online Journal of Public Health Informatics 2015;7(1):e97.

516. Austin EE. Coordinated Enhanced Surveillance with Healthcare Entities for Mass Gathering Events. Online Journal of Public Health Informatics 2017;9(1):e96.

517. Stephens E. Development of Syndrome Definitions for Acute Unintentional Drug and Heroin Overdose. Online Journal of Public Health Informatics 2017;9(1):e15.

518. Yuan CM, Love S, Wilson M. Syndromic Surveillance at Hospital Emergency Departments -- Southeastern Virginia. MMWR: Morbidity & Mortality Weekly Report 2004;53:56-8.

519. Baer A, Elbert Y, Burkom HS, Holtry R, Lombardo JS, Duchin JS. Usefulness of syndromic data sources for investigating morbidity resulting from a severe weather event. Disaster Medicine and Public Health Preparedness 2011;5(1):37-45.

520. Kwan-Gett TS, Baer A, Duchin JS. Spring 2009 H1N1 influenza outbreak in King County, Washington. Disaster Medicine and Public Health Preparedness 2009;3(Suppl. 2):S109-S16.

521. Lober WB, Baer A, Karras BT, Duchin JS. Collection and integration of clinical data for surveillance. Medinfo 2004;11:1211-5.

522. Baer A, Coberly J, Hung L, Burkom H, Loschen W, Lombardo J, et al. Classification of Emergency Department Syndromic Data for Seasonal Influenza Surveillance. Advances in Disease Surveillance 2007;4:233.

523. Baer A, Duchin J. Performance of a Syndromic Surveillance System for Detecting Carbon Monoxide Poisoning Following a Severe Windstorm. Advances in Disease Surveillance 2007;4:232.

524. Baer A, Jackson M, Duchin JS. What is the Value of a Positive Syndromic Surveillance Signal? Advances in Disease Surveillance 2007;2:192.

525. Baer A, Duchin J. Monitoring Staphylococcus Infection Trends with Biosurveillance Data. Advances in Disease Surveillance 2008;5:162.

526. Baer A, Elbert Y, Burkom H, Holtry R, Duchin J. Utility of Syndromic Surveillance for Investigating Morbidity Resulting from a Severe Weather Event. Advances in Disease Surveillance 2008;5:92.

527. Dilley JA, Baer A, Duchin J, Maher JE. Using a Syndromic Surveillance System to Evaluate the Impact of a Change in Alcohol Law. Online Journal of Public Health Informatics 2015;7(1):e70.

528. Lober WB, Trigg LJ, Karras BT, Bliss D, Ciliberti J, Stewart L, et al. Syndromic surveillance using automated collection of computerized discharge diagnoses. Journal of Urban Health 2003;80(1):i97-i106.

529. Duchin JS. Epidemiological response to syndromic surveillance signals. Journal of Urban Health 2003;80(1):i115-i6.

530. Baer A. An information visualization approach to improving data quality. Emerging Health Threats Journal 2011;4:10.

531. Rodriguez C, Baer A, Baseman J, Painter I, Yanez D, Koepsell T, et al. School absenteeism and emergency department ILI rates in King County, WA 2003–2009. Emerging Health Threats Journal 2011;4(1):s109.

532. Marsden-Haug N, Baer A, Metcalf H, Turner N, Shoemaker P, Duchin J. Syndromic Surveillance for Influenza in Washington State: A Local and Regional Perspective. Advances in Disease Surveillance 2007;4:256.

533. Karras B, Bliss D, Horn S, Metcalf H, Wagner S, BLober, et al. Syndromic Surveillance Information Collection – Geocodes for Urban to Rural Mixed Environments: SSIC-Geo. Advances in Disease Surveillance 2006;1:38.

534. Karras BT, Bliss D, Wagner S, Horn S, Lindquist S. Comparison of automated geocoding methods in a distributed multi-county disease surveillance system. Advances in Disease Surveillance 2007;2:16.

535. Turner N, Klementiev A. Integrating Early Event Detection into Local Disease Surveillance and Response. Advances in Disease Surveillance 2006;1:72.

536. Nania JM, Tansy RM. Monitoring the Vital Signs of Community Health-The Pyramid Syndromic Surveillance Project. Advances in Disease Surveillance 2007;4:58.

537. Karon AE, Heffernan RT. Google Flu Trends® versus ED Syndromic Surveillance, Wisconsin 2009. ISDS; Miami, Fl2009.

538. Foldy S, Biedrzycki PA, Barthell EN, Healy-Haney N, Baker BK, Howe DS, et al. Syndromic surveillance using regional emergency medicine Internet. Annals of Emergency Medicine 2004;44(3):242-6.

539. Foldy SL, Biedrzycki PA, Baker BK, Swain GR, Howe DS, Gieryn D, et al. The Public Health Dashboard: a surveillance model for bioterrorism preparedness. Journal of Public Health Management & Practice 2004;10(3):234-40.

540. Foldy S, Biedrzycki P, Barthell E, Haney-Healey N, Baker B, Howe D, et al. Milwaukee biosurveillance project: Real-time syndromic surveillance using secure regional internet. Journal of Urban Health 2003;80(1):i126-i.

541. Bellazzini MA, Minor KD. ED syndromic surveillance for novel H1N1 spring 2009. American Journal of Emergency Medicine 2011;29(1):70-4.

542. Marc A Bellazzini, Ronald E Gangnon, James E Svenson. Fever as a Measure for Early Detection of Influenza Outbreaks in the Emergency Department. Advances in Disease Surveillance 2008;5:4.

543. Bellazzini MA, Svenson JE. Discrete Data from Electronic Medical Records - Next Generation Data Sets for Syndromic Surveillance. Advances in Disease Surveillance 2008;5:3.

544. Kuramoto-Crawford SJ, Spies EL, Davies-Cole J. Detecting Suicide-Related Emergency Department Visits Among Adults Using the District of Columbia Syndromic Surveillance System. Public Health Reports 2017;132(1).

545. Griffin BA, Jain AK, Davies-Cole J, Glymph C, Lum G, Washington SC, et al. Early detection of influenza outbreaks using the DC Department of Health's syndromic surveillance system. BMC Public Health 2009;9:483.

546. Stoto MA, Jain A, Diamond A, Davies-Cole J, Adade A, Washington S, et al. Syndromic Surveillance System Evaluation -- District of Columbia, 2001-2004. MMWR: Morbidity & Mortality Weekly Report 2005;54:202-.

547. Lum GR, Siaway G, DeHaan K, Kidane G, Washington SC. Geospatial Direction of Syndromes in the District of Columbia. Advances in Disease Surveillance 2007;2:**57**.

548. Stoto MA, Jain A, Griffin BA, Davies-Cole JO, Lum G, Kidane G, et al. Evaluation of the DC Department of Health’s Syndromic Surveillance System. Advances in Disease Surveillance 2007;2:213.

549. Lombardo J, Burkom H, Elbert E, Magruder S, Lewis SH, Loschen W, et al. A systems overview of the electronic surveillance system for the Early Notification of Community-Based Epidemics (ESSENCE II). Journal of Urban Health 2003;80:i32-i42.

550. Begier EM, Sockwell D, Branch LM, Davies-Cole JO, Jones LH, Edwards L, et al. The National Capitol Region's emergency department syndromic surveillance system: do chief complaint and discharge diagnosis yield different results? Emerging Infectious Diseases 2003;9(3):393-6.

551. Brown H, Kistner K, Zapata I, Romanosky A. Emergency Department Visits for Influenza-Like Illness and Over the Counter Sales of Flu Remedies in the National Capitol Region, 2003-2007. Advances in Disease Surveillance 2007;4:236.

552. Chu A, Blythe D, Tolson K, Collier D, Minson M. Identifying a Meningitis Case through Syndromic Surveillance: An Example of Detecting Events of Public Health Importance and Improving Situational Awareness. Advances in Disease Surveillance 2007;2:98.

553. Aslam S, , Brown H, , Ajit I, Adams S. Syndromic Surveillance for Historical 2009 Presidential Inauguration. ISDS; Miami, Fl 2009.

554. Zhang Y, May L, Stoto M. Evaluating University syndromic surveillance systems during the 2009 H1N1 influenza pandemic. Emerging Health Threats Journal 2011;4(1):s123.

555. Walsh A. Comparing Emergency Department Gunshot Wound Data with Mass Casualty Shooting Reports. Online Journal of Public Health Informatics 2017;9(1):e33.

556. Walsh A. Going Beyond Chief Complaints to Identify Opioid-Related Emergency Department Visits. Online Journal of Public Health Informatics 2017;9(1):e39.

557. Kaydos-Daniels SC, Rojas Smith L, Farris TR. Biosurveillance in outbreak investigations. Biosecurity and bioterrorism: Biodefense Strategy, Practice, and Science 2013;11(1):20-8.

558. Hiller KM, Stoneking L, Min A, Rhodes SM. Syndromic surveillance for influenza in the emergency department - a systematic review. PLoS ONE 2013;8(9):e73832.

559. Buckeridge DL. Outbreak detection through automated surveillance: a review of the determinants of detection. Journal of Biomedical Informatics 2007;40(4):370-9.
